# The status of cryptococcosis in Latin America

**DOI:** 10.1590/0074-02760170554

**Published:** 2018-04-05

**Authors:** Carolina Firacative, Jairo Lizarazo, María Teresa Illnait-Zaragozí, Elizabeth Castañeda, Latin American Cryptococcal Study Group

**Affiliations:** 1 University of Sydney, Sydney Medical School, Westmead Hospital, Molecular Mycology Research Laboratory, Sydney, Australia; 2 Universidad de Pamplona, Hospital Universitario Erasmo Meoz, Internal Medicine Department, Cúcuta, Colombia; 3 Tropical Medicine Institute Pedro Kourí, Bacteriology-Mycology Department Research, Diagnosis and Reference Centre, Havana, Cuba; 4 Instituto Nacional de Salud, Bogotá, Colombia; 5 Latin American Cryptococcal Study Group

**Keywords:** Cryptococcus neoformans, Cryptococcus gattii, Latin America, cryptococcosis

## Abstract

Cryptococcosis is a life-threatening fungal infection caused by the encapsulated yeasts *Cryptococcus neoformans* and *C. gattii*, acquired from the environment*.* In Latin America, as occurring worldwide, *C. neoformans* causes more than 90% of the cases of cryptococcosis, affecting predominantly patients with HIV, while *C. gattii* generally affects otherwise healthy individuals. In this region, cryptococcal meningitis is the most common presentation, with amphotericin B and fluconazole being the antifungal drugs of choice. Avian droppings are the predominant environmental reservoir of *C. neoformans*, while *C. gattii* is associated with several arboreal species. Importantly, *C. gattii* has a high prevalence in Latin America and has been proposed to be the likely origin of some *C. gattii* populations in North America. Thus, in the recent years, significant progress has been made with the study of the basic biology and laboratory identification of cryptococcal strains, in understanding their ecology, population genetics, host-pathogen interactions, and the clinical epidemiology of this important mycosis in Latin America.

In Latin America, the study of cryptococcosis and its etiological agents has become increasingly important, as this mycosis has significant morbidity and mortality, with more than 5,000 individuals affected with cryptococcal meningitis each year, and 2,400 attributable annual deaths in Latin America alone. The main goal of this review is to present recent and relevant data regarding the studies done in Latin American countries on cryptococcosis and the etiological agents *Cryptococcus neoformans*, and *C. gattii*. Topics related to epidemiology, clinical data and treatment, molecular studies on clinical and environmental isolates, host-pathogen interactions, laboratory data on antifungal susceptibility, preliminary data detecting antigenemia in patients, and studies to search for the environmental niche of the fungus are presented here.

*Epidemiology* - *C. neoformans* is the most important cause of fungal meningitis in the world, and in sub-Saharan Africa, meningeal cryptococcosis is the most common type of meningitis in adults infected with HIV (Rajasingham et al. 2017). From a total of 223,100 cases of meningeal cryptococcosis that were estimated to have occurred globally in people living with HIV in 2014, the third largest number of cases in the world were from Latin America, with an estimated incidence of 5,300 cases per year. From those, Brazil and Colombia were the countries with the highest incidence, between 1,001 to 2,500 cases, followed by Argentina and Mexico with an incidence of 501 to 1,000 cases (Rajasingham et al. 2017). In particular, an average annual incidence of 4.5 cases of meningeal cryptococcosis per 10^6^ inhabitants was reported in the general population of the state of Rio de Janeiro, Brazil (Leimann and Koifman 2008). In Colombia, the average annual incidence of cryptococcosis in the general population has been estimated to be 2.4 cases per 10^6^ inhabitants, while in the HIV-infected population this value has been estimated to be 3,000-3,300 cases per 10^6^ people (Lizarazo et al. 2007, Escandón et al. 2012). In addition, Lizarazo et al. (2014a) reported an average annual incidence of 0.0017 cases per 10^6^ in Colombian children.

There is little data regarding the prevalence of cryptococcosis in Latin America. In Mexico, a study of the etiological agents of meningoencephalitis found that the most common mycosis was cryptococcosis, with a prevalence of 10% (Barriga et al. 2005). In Venezuela, a study of systemic mycoses performed at a national reference centre found that cryptococcosis ranked third with a prevalence of 19%, following histoplasmosis and paracoccidioidomycosis; however, in the population with HIV, *C. neoformans* was isolated in 27% of the cases, following the etiological agent of histoplasmosis (Reviákina et al. 2007). A national survey conducted in Argentina identified cryptococcosis as the second most frequent deep fungal infection with a prevalence of 20%, following that of yeast fungemia (Davel and Canteros 2007). In Mexico, *C. neoformans* had a prevalence of 21% causing fungemia in patients with different types of immunosuppression, only following *Histoplasma capsulatum* (Gaona-Flores et al. 2016). In the same country, cryptococcosis was the third most common invasive fungal infection (13%) found in a highly specialised hospital, following candidiasis and mucormycosis (Méndez-Tovar et al. 2016). In Colombia, Castro-Jiménez et al. (2011) reported cryptococcosis as the most common opportunistic mycosis in patients with HIV/AIDS, with a prevalence of 76%. In the same population in Guatemala, it was estimated that meningeal cryptococcosis is the fourth most common opportunistic fungal infection following candidiasis (esophageal), *Pneumocystis jirovecii* pneumonia, and disseminated histoplasmosis (Medina et al. 2017).

In Colombia, among opportunistic infections of the central nervous system (CNS) in patients with HIV, cryptococcosis is the second most common, after toxoplasmosis, which has been demonstrated in clinical studies (Lizarazo et al. 2006, Ávila and González 2007), and autopsies (Mantilla and Cárdenas 2009). The same findings have been described in Cuba (Lamotte 2014).

Most cases of cryptococcosis are reported in young HIV positive male patients. Among the HIV negative patients, males are also the predominant sex, although in a smaller proportion, and patients also tend, on average, to be slightly older (Dromer et al. 1996). This epidemiological distribution has also been found in Latin America (Table I). Globally, as in many developing countries, many cases of cryptococcosis in Latin America may not be reported or be confirmed by diagnosis, which in part is reflected by the number of people living with AIDS not in care, undiagnosed, and lost to follow-up, or living in resource-limited areas, which does not allow for a prompt and correct diagnosis (Rajasingham et al. 2017).

**TABLE I:** Cryptococcosis in Latin America. Epidemiological and clinical data

Country	Number of cases	Percentage of cases									Reference

		Male	HIV+	Clinical presentation (%)	Headache	High ICP > 25 cm H_2_0	*C. gattii*	Lethality			

								global	HIV+	HIV-	
Argentina											
Buenos Aires	58	100	100	Meningitis (100)	88			21	21		Cangelosi et al. (2009)
Buenos Aires	2041	75	98	Meningitis (90)	70	79	0.3		40		Arechavala et al. (2017)
Buenos Aires	58	88	100	Meningitis (100)				19	19		Ramírez et al. (2017)
Buenos Aires Province	106	71	100	Neuro (91)				36	36		Mónaco and Antabak (2008)
Chaco (Corrientes)	26	69	69	Meningitis (96)			0				Cattana et al. (2015)
Córdoba (Córdoba)	53	66	100								Luque et al. (2017)
Brazil											
Bahia	104	62	12		93			43	12		Darzé et al. (2000)
Bahia	50	70	52	Meningitis (100)	92	77 (> 20 cmH_2_O)		22			Mascarenhas-Batista et al. (2013)
Goiás (Goiânia)	62	74	85	Meningitis (100)	76		6	48	47	56	Hasimoto e Souza et al. (2013)
Mato Grosso do Sul (Campo Grande)	123	68	84	Neuro (84)	76		10	50	51	41	Lindenberg et al. (2008)
Mina Gerais (Uberlandia)	96	80	81	Meningitis (56)			7	73	77		Moreira et al. (2006)
Mina Gerais (Uberlandia)	41	83	85	Meningitis (75)			0	58	60	25	Aguiar et al. (2017)
Mina Gerais (Uberaba)	131	69	91	Meningitis (79)	60	53	5	51			Mora et al. (2012)
Río de Janeiro (Rio de Janeiro)	696	73	61	Meningitis (96)				52	61		Leimann and Koifman (2008)
Rio de Janeiro (Rio de Janeiro)	138	76	100	Meningitis (100)				62	62		Ramírez et al. (2017)
Rio Grande do Sul (Porto Alegre)	126	77	95	Meningitis (100)	80		5				Leal et al. (2008)
São Paulo (São Paulo)	98	78	97	Meningitis (100)	91	55		30			Vidal et al. (2012)
São Paulo (São Paulo)	29	55	0	Neuro (86)	96	76 (≥ 20 cmH_2_O)	64	21		21	Lomes et al. (2016)
National, children	53	53	24	Neuro (96)	85		30	41			Severo et al. (2009)
Bolivia											
Cochabamba	31	83	100	Meningitis (100)	23			67	67		Castro and Córdoba (2014)
Chile											
Santiago	28	93	100	Meningitis (100)				21	21		Ramírez et al. (2017)
Colombia											
National 1997-2005	931	83	78	Neuro (96)	85		4				Lizarazo et al. (2007)
National 2006-2010	526	77	83	Neuro (81)	84	20	3	32			Escandón et al. (2012)
Norte de Santander (Cúcuta)	90	77	70	Meningitis (100)	90	57	22	34	49	16	Lizarazo et al. (2012)
Atlántico (Barranquilla)	41	76	78	Meningitis (66)			1				Noguera et al. (2015)
National, children	41	58	24	Meningitis (88)	78		6				Lizarazo et al. (2014a)
Costa Rica											
San José	71	79	73	Neuro (73)	100		0	46	46	0	Ávila and Villalobos (2016)
Cuba											
La Habana	16	82	0	Neuro (100)	81			31		31	Fernández-Concepción et al. (2003)
Ecuador											
Guayaquil	82	73	100	Meningitis (100)	78						Sánchez et al. (2016)
Guatemala											
Guatemala	110	73	100	Meningitis (100)	80			14	14		Romero et al. (2005)
Guatemala	60	80	100	Meningitis (100)				43	43		Mejía and del Valle (2012)
Guatemala	28	72	100	Meningitis (100)	93			21	21		Herrera et al. (2014)
Honduras											
Tegucigalpa	27	59	100	Meningitis (100)				33	33		Ramírez et al. (2017)
Mexico											
Ciudad de Mexico	34	85	100	Meningitis (100)				38	38		Ramírez et al. (2017)
Panama											
Panama	28	61	100	Meningitis (100)	96	57 (≥ 20 cmH_2_O)		50	50		González et al. (2013)
Paraguay											
Itauguá	13	76	62	Meningitis (100)	90			46	83	20	Sierra (2013)
Peru											
Lima	47	87	100	Meningitis (100)	100	64 (≥ 20 cmH_2_O)	0	19	19		Dammert et al. (2008)
Lima	90	77	100	Meningitis (100)				41	41		Canessa et al. (2011)
Lima	234		100	Meningitis (100)	86			20	20		Concha-Velasco et al. (2017)
Uruguay											
Montevideo	147		92				0.7				Carbia et al. (2017)
Venezuela											
Caracas	110	83	83	Meningitis (97)				30	32	21	Pérez et al. (2009)

ICP: intracranial pressure; Neuro: meningitis, meningoencephalitis and cryptococcoma.

*Clinical aspects* - Cryptococcosis mainly affects the CNS, causing meningitis. In a lesser proportion it affects the lungs, and organs such as skin, eyes, prostate, and bone, among others (Maziarz and Perfect 2016). Classically, patients with meningeal cryptococcosis present a clinical picture consisting of headache and fever, lasting approximately two weeks. Many of these patients also present with nausea, vomiting, cranial nerve involvement, and decreased visual acuity due to intracranial hypertension. If the disease progresses without treatment, mental changes, seizures, and a decreased state of consciousness leading to coma are observed (Limper et al. 2017, Williamson et al. 2017). In Latin America, meningeal cryptococcosis is also the main form of clinical presentation, with headache as the cardinal symptom (Table I). Intracranial hypertension, one of the most feared complications, has been reported in several Latin American studies, in more than 50% of the cases (Table I), which suggests a high percentage of patients with advanced forms of the disease. In autopsy studies conducted in Latin America, patients with neurocryptococcosis, most of whom were infected with HIV, predominantly had disseminated forms of the disease, with multiple organ involvement. Pure meningoencephalitic forms are less frequent, and the presence of cryptococcomas very rare (Reséndiz et al. 2008, Klock et al. 2009, Mantilla and Cárdenas 2009, Torres et al. 2016).

*Treatment* - For meningeal cryptococcosis, the treatment of choice recommended by the guidelines of the Infectious Diseases Society of America (IDSA), consists of a combination of amphotericin B deoxycholate (AmBd) 0.7 mg/kg/day, and 5-fluorocytosine (5FC) 100 mg/kg/day for two weeks as induction phase, followed by fluconazole (FCZ) 400 mg daily for eight weeks as consolidation phase, and FCZ 200 mg daily as the maintenance phase. In the induction phase it is recommended to use, as an alternative, the use of liposomal AmB (LAmB) associated (or in combination) with 5FC (Perfect et al. 2010). In Latin America, due to the unavailability of 5FC and the high cost of LAmB preparations, an alternative regimen recommended by the IDSA for countries with limited resources, with a good level of evidence (AI), consists of AmBd 0.7 mg/kg/day, plus FCZ 800 mg/day for the induction phase of two weeks; followed by FCZ 800 mg per day for eight weeks for the consolidation phase (Table II). An alternative scheme for the induction phase is the use of AmBd alone, at a dose greater than 1 mg/kg/day (Perfect et al. 2010). Similar considerations have been made in the Brazilian (Moretti et al. 2008), and Colombian (Castañeda and Lizarazo 2012) management guidelines.

**TABLE II:** Cryptococcosis in Latin America. Clinical presentation and treatment

Country	Number of cases	Percentage of cases							Reference

		Clinical presentation (%)	AmB	AmB + FCZ	AmB + 5FC	AmB + FCZ or 5FC	FCZ	Other	
Argentina									
Buenos Aires	2041	Meningitis (90)	100 (1996-2009)	100 from 2010	100 (1986-1995)				Arechavala et al. (2017)
Provincia de Buenos Aires	106	Neuro (91)		100					Mónaco and Antabak (2008)
Brazil									
Bahia (Salvador)	50	Meningitis (100)	62	32	0		4	2	Mascarenhas-Batista et al. (2013)
Mato Grosso do Sul (Campo Grande)	123	Neuro (84)	86				60^*a*^		Lindenberg et al. (2008)
Mina Gerais (Uberlandia)	96	Meningitis (56)	54	19	0		3	3	Moreira et al. (2006)
Mina Gerais (Uberlandia)	41	Meningitis (75)	85				75^*a*^		Aguiar et al. (2017)
São Paulo (São Paulo)	29	Neuro (86)				100 including LAmB		3	Lomes et al. (2016)
National, children	32		28			66			
Bolivia									
Cochabamba	31	Meningitis (100)	100						Castro and Córdoba (2014)
Colombia									
National 1997-2005	931	Neuro (96)	73	21				6	Lizarazo et al. (2007)
National 2006-2010	526	Neuro (81)	72	28					Escandón et al. (2012)
Norte de Santander (Cúcuta)	90	Meningitis (100)		93					Lizarazo et al. (2012)
Atlántico	41	Meningitis (66)	29	5			17		Noguera et al. (2015)
National, children	41	Meningitis (88)	61	16	6	3	3	9	Lizarazo et al. (2014a)
Costa Rica									
San José	71	Cerebral (73)	79	21					Ávila and Villalobos (2016)
Cuba									
La Habana	16	Neuro (100)	69	23		8			Fernández-Concepción et al. (2003)
Guatemala									
Guatemala	110	Meningitis (100)	78 (15) *				6		Romero et al. (2005)
Panamá									
Panamá	28	Meningitis (100)	100						González et al. (2013)
Paraguay									
Itauguá	13	Meningitis (100)	85	7					Sierra (2013)
Peru									
Lima	47	Meningitis (100)		100					Dammert et al. (2008)
Lima	234	Meningitis (100)	62	25				13	Concha-Velasco et al. (2017)

*a*: consolidation phase; AmB: amphotericin B; *LAmB: liposomal amphotericin B; FCZ: fluconazole; 5FC: 5 fluocytosine; neuro: meningitis, meningoencephalitis and cryptococcoma.

The lethality of meningeal cryptococcosis in Latin America has been reported to range from 13% to 73%, with many cases ranging between 30% and 60% (Table I), which are very high figures compared to that of the industrialised countries (van der Horst et al. 1997), but similar to those recorded in sub-Saharan Africa (Jarvis and Harrison 2016). In Brazil, among the systemic mycoses, cryptococcosis causes the highest number of deaths in patients with HIV (Prado et al. 2009). In this country, the mortality rate of cryptococcal infections was 0.47 per million inhabitants, and was the thirteenth cause of death (Soares 2015). In Colombia, however, the survival rate of patients with meningogenic cryptococcosis has been reported to be 82% for HIV negative patients, and only 46% for HIV positive patients, with a survival time of only four months after diagnosis (Lizarazo et al. 2012).

*Molecular studies on C. neoformans and C. gattii* - Several broadly used molecular techniques have been used to determine the genotype of individual isolates, and compare groups of isolates among *C. neoformans* and *C. gattii* species, to carry out molecular epidemiology and population genetics studies. Among these techniques, polymerase chain reaction (PCR) fingerprinting (Meyer and Mitchell 1995), amplified fragment length polymorphisms (AFLP) (Boekhout et al. 2001, Hagen et al. 2010), microsatellite typing (Illnait-Zaragozi et al. 2010a), restriction fragment length polymorphisms (RFLP) (Meyer et al. 2003), multilocus sequence typing (MLST) (Meyer et al. 2009), and more recently, whole genome sequencing (WGS) (Loftus et al. 2005, D’Souza et al. 2011), have allowed for the identification of eight major molecular types among the cryptococcal species in the world: VNI (AFLP1) and VNII (AFLP1A/B) for *C. neoformans* var. *grubii* serotype A isolates, VNIV (AFLP2) for *C. neoformans* var. *neoformans* serotype D isolates, VNIII (AFLP3) for hybrids between the serotypes A and D, and VGI (AFLP4), VGII (AFLP6), VGIII (AFLP5) and VGIV (AFLP7) for *C. gattii* serotype B, and C isolates.

Although in most of the studies on *Cryptococcus* and cryptococcosis reported from Latin America, the isolates have been identified only at species level, variety, or serotype; it is now possible to determine the major molecular type of the cryptococcal isolates recovered not only from clinical, but also from environmental sources, and much less frequently from veterinary samples, by using one or more of the above mentioned molecular techniques.

Using these techniques, the major molecular type of 3,486 isolates in Latin America is presently known, with most of the isolates reported from Brazil (45.87%), followed by Colombia (24.99%), Mexico (9.21%), Argentina (8.09%), Cuba (6.05%), Venezuela (2.90%), Peru (1.06%), Ecuador (0.77%), Chile (0.55%), Guatemala (0.43%), as well as Honduras, Paraguay, and Uruguay with one isolate each (Table III). From Bolivia, Costa Rica, Dominican Republic, El Salvador, Haiti, Nicaragua, and Panama, there are not data reported about the molecular type of cryptococcal strains.

**TABLE III:** Distribution of *Cryptococcus neoformans* and *C. gattii* isolates recovered in Latin America and identified at molecular type level

Country*	n	Source	*C. neoformans*				*C. gattii*				Total
	
			VNI	VNII	VNIII	VNIV	VGI	VGII	VGIII	VGIV	
Brazil		Clinical	695	38	-	-	19	269	18	-	1039
	1599	Environmental	301	8	1	23	7	215	-	-	555
		Veterinary	-	-	-	-	-	5	-	-	5
Colombia	871	Clinical	317	17	-	6	10	54	39	2	445
		Environmental	229	1	-	-	3	106	76	11	426
Mexico	321	Clinical	209	21	12	7	11	5	26	7	298
		Environmental	18	-	-	-	-	-	5	-	23
Argentina	282	Clinical	209	13	9	2	3	1	1	-	238
		Environmental	19	-	-	-	23	-	2	-	44
Cuba	211	Clinical	141	-	-	-	-	-	1	-	142
		Environmental	68	-	-	-	-	-	-	-	68
		Veterinary	-	-	-	-	1	-	-	-	1
Venezuela	101	Clinical	76	6	1	-	5	10	3	-	101
Peru	37	Clinical	25	9	-	2	1	-	-	-	37
Ecuador	27	Clinical	27	-	-	-	-	-	-	-	27
Chile	19	Clinical	4	3	3	5	-	-	-	-	15
		Environmental	4	-	-	-	-	-	-	-	4
Guatemala	15	Clinical	14	-	-	-	-	-	1	-	15
Honduras	1	Clinical	-	-	-	-	1	-	-	-	1
Paraguay	1	Clinical	-	-	-	-	-	-	1	-	1
Uruguay	1	Environmental	-	-	-	-	-	1	-	-	1

Total		Clinical	1,717	107	25	22	50	339	90	9	2,359
	3,486	Environmental	639	9	1	23	33	322	83	11	1,121
		Veterinary	-	-	-	-	1	5	-	-	6

Molecular data have been combined from studies reported in **Argentina** (Meyer et al. 2003, Refojo et al. 2009, Cattana et al. 2013, Mazza et al. 2013, Cattana et al. 2014, Cattana et al. 2015, Cicora et al. 2015, Arechavala et al. 2017), **Brazil** (Casali et al. 2003, Trilles et al. 2003, Igreja et al. 2004, Abegg et al. 2006, Ribeiro et al. 2006, Matsumoto et al. 2007, Lugarini et al. 2008, Ribeiro & Ngamskulrungroj 2008, dos Santos et al. 2008, Trilles et al. 2008, Costa et al. 2009, Andrade-Silva et al. 2010, Pinto Jr et al. 2010, Souza et al. 2010, Ferreira-Paim et al. 2011, d, da Silva et al. 2012, Freire et al. 2012, Silva et al. 2012, Cardoso et al. 2013, Tsujisaki et al. 2013, Anzai et al. 2014, Nascimento et al. 2014, Brito-Santos et al. 2015, Headley et al. 2015, Castro e Silva et al. 2016, Figueiredo et al. 2016, Lomes et al. 2016, Souto et al. 2016, Aguiar et al. 2017, d, da Silva et al. 2017), **Chile** (Calvo et al. 2001, Meyer et al. 2003, Solar et al. 2015), **Colombia** (Meyer et al. 2003, Escandón et al. 2006, Escandón et al. 2010, Firacative et al. 2011, Lizarazo et al. 2012, Lizarazo et al. 2014a, Lizarazo et al. 2014b, Noguera et al. 2015, Cogliati et al. 2016, Firacative et al. 2016, Noguera et al. 2017, Vélez and Escandón 2017), **Cuba** (Illnait-Zaragozi et al. 2010a, Illnait-Zaragozi et al. 2010b, Illnait-Zaragozi et al. 2011b, Illnait-Zaragozi et al. 2013), **Ecuador** (Sánches et al. 2017), **Guatemala** (Meyer et al. 2003), **Honduras** (Boekhout et al. 2001), **Mexico** (Meyer et al. 2003, Olivares et al. 2009, Firacative et al. 2016, González et al. 2016), **Paraguay** (Firacative et al. 2016), **Peru** (Meyer et al. 2003, Bejar et al. 2015), **Uruguay** (Engelthaler et al. 2014) and **Venezuela** (Calvo et al. 2001, Meyer et al. 2003, Firacative et al. 2016, Ferrara et al. 2017). *From Bolivia, Costa Rica, Dominican Republic, El Salvador, Haiti, Nicaragua, and Panama there is not data reported about the molecular type of cryptococcal strains.

From these 3,486 isolates, 67.7%, and 32.2% were recovered from clinical, and environmental sources, respectively. In concordance with global data, the molecular type VNI (AFLP1), corresponding to *C. neoformans* var. *grubii*, serotype A is the predominant molecular type (72.8%) causing cryptococcosis in Latin America, followed by *C. gattii* molecular type VGII (AFLP6) (14.4%) (Fig. 1). Interestingly, in the environment these two molecular types also predominate with slightly different proportions (57.0% for *C. neoformans* VNI, and 28.7% for *C. gattii* VGII) (Fig. 1), although this distribution may be biased by the high prevalence of *C. gattii* VGII in Brazil and Colombia, which are the countries with the largest number of studied isolates (Fig. 1, Table III). Of all these studies, only six *C. gattii* isolates (0.2%) have been reported from veterinary cases: one molecular type VGI from Cuba (Illnait-Zaragozi et al. 2011b) and five molecular type VGII from Brazil (Cardoso et al. 2013, Headley et al. 2015, da Silva et al. 2017) (Fig. 1). Interestingly, in Brazil an outbreak of infection occurred in a breeding aviary in São Paulo, where seven parrot-like birds died of disseminated cryptococcosis. Although the molecular type of the causative agent was not identified, all cases were caused by *C. gattii* serotype B, resistant to FCZ, as determined by biochemical, physiological, and serological testing (Raso et al. 2004). To date, veterinary cases of *C. neoformans* have not yet been reported in Latin American countries.

**Fig. 1:**
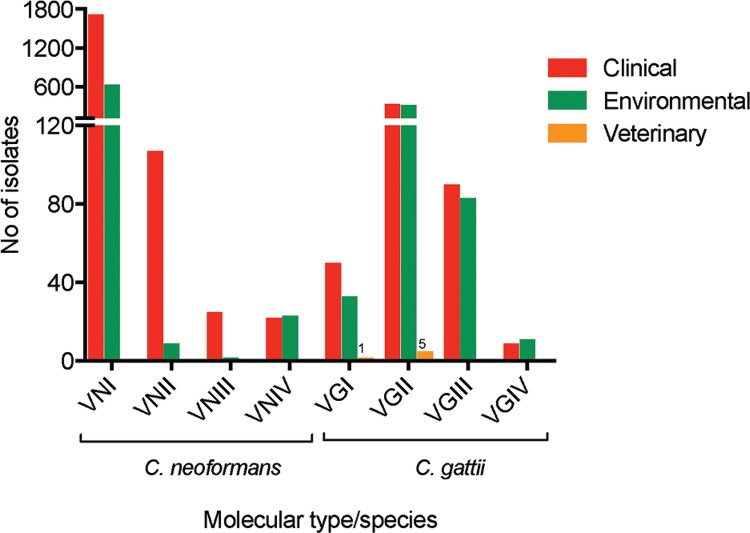
: distribution of *Cryptococcus neoformans* and *C. gattii* identified at molecular type level (n = 3486), recovered from clinical, environmental*a* or veterinary sources in Latin America. Molecular data have been combined from studies reported in Argentina, Brazil, Chile, Colombia, Cuba, Ecuador, Guatemala, Honduras, Mexico, Paraguay, Peru, Uruguay, and Venezuela (Table III). Data from Bolivia, Costa Rica, Dominican Republic, El Salvador, Haiti, Nicaragua, and Panama about the molecular typing of cryptococcal strains has not been reported. *a*: *C. neoformans* has been mostly recovered from avian droppings, decaying organic matter, and soil (Casali et al. 2003, Ribeiro et al. 2006, Lugarini et al. 2008, Ribeiro and Ngamskulrungroj 2008, Andrade-Silva et al. 2010, Illnait-Zaragozi et al. 2010a, Souza et al. 2010, Ferreira-Paim et al. 2011, Canónico-González et al. 2013, Castro e Silva et al. 2016). *C. gattii* is associated with several tree species (Escandón et al. 2006, Trilles et al. 2008, Costa et al. 2009, Refojo et al. 2009, Escandón et al. 2010, Firacative et al. 2011, Mazza et al. 2013, Anzai et al. 2014, Cattana et al. 2014, Brito-Santos et al. 2015, Vélez and Escandón 2017).

Clinical cases of cryptococcosis from which the major molecular type of the isolates has been identified, date back to 1961 (Meyer et al. 2003), with most of the studies being retrospective. While Argentina, Brazil, Colombia, and Cuba have described isolates from the 1980s (Igreja et al. 2004, Escandón et al. 2006, Illnait-Zaragozi et al. 2010a, Arechavala et al. 2017), most of the isolates recovered in Latin America are from the mid-1990s, to date. Case reports, in which *C. gattii* has been isolated as etiological agent, have been documented much more recently (Illnait-Zaragozi et al. 2013, Nascimento et al. 2014, Cicora et al. 2015, Solar et al. 2015, Noguera et al. 2017), with the earliest occurring in 2005 in Brazil (Pinto Jr et al. 2010). The major molecular type of the environmental isolates was first reported about two decades ago in Cuba (Illnait-Zaragozi et al. 2010a), although most of the reports on the molecular types of *C. neoformans* and *C. gattii* from the environment are from after the year 2000.

The combined analysis of the molecular data also showed that in Colombia, the prevalence of the *C. gattii* VGIII is very similar to that of C. *gattii* VGII and that in Mexico, the molecular type VGIII is the most common among *C. gattii* isolates and the second most common after *C. neoformans* VNI. In Argentina however, the most common molecular type among *C. gattii* isolates is VGI (Fig. 2). Mexico is the only country where all major molecular types have been reported, while in Brazil and Argentina only *C. gattii* VGIV seems to be absent and in Colombia only *C. neoformans* VNIII has not been recognised. However, apart from VNIII, the hybrid most commonly found in *C. neoformans*, inter- and intra-specific hybrids have been reportedly for Latin America. One isolate described as the VNII/VNIV hybrid was reported from Argentina, another isolate with the same genotype VNII/VNIV and two isolates VNI/VGII from Brazil, and five isolates VNI/VNII and one isolate VNI/VGII from Colombia (Aminnejad et al. 2012, Aminnejad et al. 2016).

**Fig. 2:**
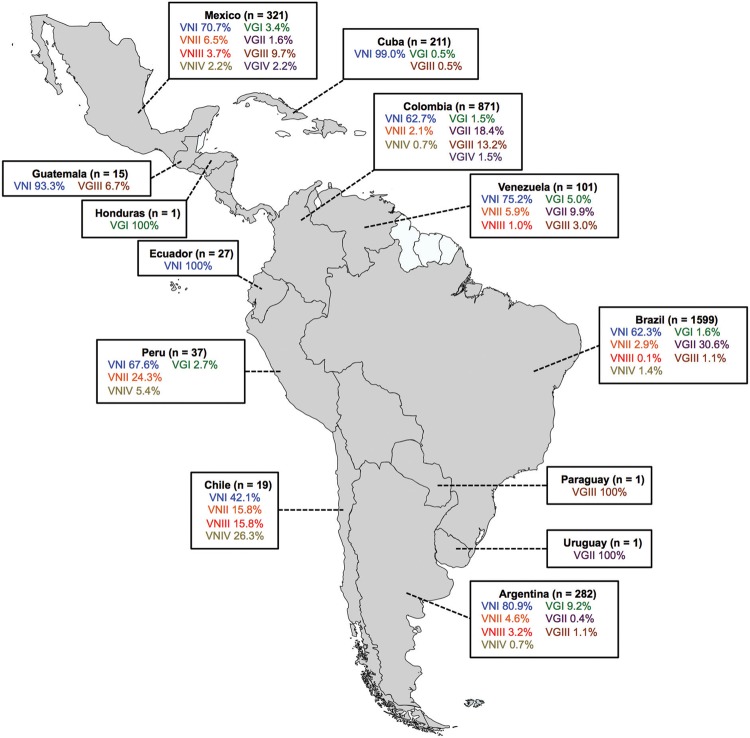
: geographic distribution of *Cryptococcus neoformans* and *C. gattii* isolates from Latin America, identified at the molecular type level (n = 3486). Molecular data have been combined from studies reported in Argentina, Brazil, Chile, Colombia, Cuba, Ecuador, Guatemala, Honduras, Mexico, Paraguay, Peru, Uruguay, and Venezuela (Table III). Data from Bolivia, Costa Rica, Dominican Republic, El Salvador, Haiti, Nicaragua, and Panama regarding the molecular typing of cryptococcal strains have not been reported.

When the mating type of the isolates was identified by specific PCR, the mating alpha was the most common in both *C. neoformans* and *C. gattii* isolates recovered in Latin America, as noted globally (Kwon-Chung et al. 2014). However, in Colombia all *C. gattii* VGIII isolates recovered from *Corymbia ficifolia* detritus, and one VGI isolate recovered from *Ficus* spp. were mating type a (Escandón et al. 2010, Firacative et al. 2011), as well as 13 clinical and three environmental VGII isolates from Brazil (Brito-Santos et al. 2015, Souto et al. 2016), and two VGIII clinical isolates recovered in Mexico (Firacative et al. 2016). Interestingly, there are no reports on *C. neoformans* mating type a in the region.

The use of MLST and WGS to further advance the understanding of *C. neoformans* and *C. gattii* populations has also been achieved in Latin America. Firstly, a highly clonal population structure of clinical and environmental isolates of *C. neoformans* var. *grubii* was revealed in Brazil (Ferreira-Paim et al. 2017), which is in agreement with the clonal evolution and dispersion of *C. neoformans* that has been reported worldwide, these results detail the absence or restriction of genetic recombination, and the persistence of widespread clones. Opposing this, the genetic population structure of many isolates of the less common *C. neoformans* var. *neoformans*, from different geographical origin, including Colombia, and from different sources, showed that this is a highly recombinant population, with the isolates being strictly correlated to each other, and characterised by high variability (Cogliati et al. 2016).

The global diversity of a number of *C. gattii* VGII isolates was also studied, which provided evidence on the evolution of this pathogen in North America and gave support to the extensive evolution in, and dispersal from, South America, most likely from the Amazonia and the Northeastern regions of Brazil, where *C. gattii* VGII strains have shown to have the most genetic variability, compared to isolates from the rest of the world (Engelthaler et al. 2014, Souto et al. 2016).

Additionally, Mexico and Colombia have been proposed to be the likely origins of the VGIII *C. gattii* population, as isolates from these countries are the basal group of isolates recovered worldwide; they are very variable genetically, and have both mating types, which may lead to sexual reproduction and recombination events (Firacative et al. 2016).

Other molecular techniques employed in Latin American countries have allowed the identification and characterisation of cryptococcal isolates, although without determining the major molecular types. PCR fingerprinting with GACA_4_ primers was used for the genotyping of the first environmental isolate of *C. gattii* recovered in Argentina (Meyer and Mitchell 1995, Davel et al. 2003). Electrophoretic karyotyping of 40 *C. neoformans*, and 11 *C. gattii* clinical isolates from Brazil, Chile and Venezuela showed a broad genotypic diversity among the isolates (Calvo et al. 2001). Sequencing of the internal transcribed spacer (ITS) identified one *C. gattii* isolate recovered from an immunocompetent patient in Chile (Solar et al. 2015), and one and five *C. neoformans* environmental isolates recovered from an almond tree in Cuba (Illnait-Zaragozi et al. 2012), and derived from pigeon excreta in Mexico (Canónico-González et al. 2013), respectively. In addition, ITS sequencing allowed for the discrimination of an uncommon species causing cryptococcosis, *C. liquefaciens*, in a HIV positive patient from Guatemala (Conde-Pereira et al. 2015). Lastly, using comparative genome hybridisation, the genome content of a clinical *C. neoformans* VNI strain from Argentina was examined, which showed that this strain shared most, if not all, of its genome with H99, the reference strain of *C. neoformans* var. *grubii* (Hu et al. 2008).

Diagnostic and identification of cryptococcal species from clinical and environmental samples has also been possible by using molecular methodologies, some of which have been developed in Latin America. PCR of the capsular gene *CAP59,* followed by RFLP was used in Argentina to determine the serotypes of cryptococcal strains directly from a yeast suspension, thus avoiding DNA extraction and making serotype identification quick, simple and inexpensive (Siachoque et al. 2010). In Colombia, a nested PCR targeting the ITS region was developed for the diagnosis of *C. neoformans* and *C. gattii* from human samples, including bronchoalveolar lavage (BAL), bronchial lavage (BL), biopsy, and, cerebrospinal fluid (CSF), showing a sensitivity and specificity of 100% (Rivera et al. 2015). In addition, in Colombia, two techniques to extract cryptococcal DNA from contaminated soil were standardised, namely glass beads with agarose blocks and an ultra-clean DNA soil kit (MoBio, Solano Beach, CA, USA), which allowed a faster yet specific detection of cryptococcal isolates from soil samples through the amplification of species-specific DNA using the primers CN4 and CN5 (Castañeda et al. 2004). The rapid, sensitive and specific identification of the eight major molecular types, including hybrid strains, from culture and directly from clinical samples is also now possible after the development of a hyperbranched rolling circle amplification of the phospholipase gene *PLB1* in combination with a semi-nested PCR (Trilles et al. 2014).

Thus, the use of molecular techniques for the study of the etiological agents of cryptococcosis in Latin America, as elsewhere, shows an undoubted value especially for rapid detection and genetic epidemiology studies, and at the same time shows that these methodologies are complementary to traditional techniques.

*Host-Cryptococcus* - The role of *C. neoformans* and *C. gattii* as the major causative agents of fungal meningoencephalitis in humans and animals worldwide (Rajasingham et al. 2017) has stimulated more than half-century’s worth of investigations on these fascinating and versatile fungi. Nevertheless, many questions about this species and the interactions with the host are not yet fully elucidated, including: (1) whether reactivation of long-term latent infection is a more important cause of cryptococcosis in patients than *de novo* acquisition from the environment; (2) the bases of the preference of some genotypes for certain hosts (i.e. immunosuppressed vs. immunocompetent) and target organs (i.e. brain vs. lung); (3) the mechanism by which the infection becomes persistent and/or recurrent despite that *in vitro* resistance to antifungal drugs does not seem to be a problem for cryptococcal treatment; and (4) the diverse pathways related to sexual reproduction, among many others (Rodrígues et al. 2007).

In Latin American, specifically in Argentina, Brazil and Cuba, different investigations related with these topics have been conducted mostly in order to reveal and comprehend host-pathogen interactions, to investigate the role of interactions between the yeasts and other microorganisms, and to review alternative approaches to antifungal treatments against cryptococcosis and other mycosis. A clear majority of the authors have attempted then to understand the role of the immune cells and the cytokines/antibodies induction not only from human clinical samples but also by using *in vitro* and *in vivo* models.

In AIDS patients with cryptococcal meningitis, cytokine patterns in the CSF and sera were shown to be related to fungal burden and clinical outcome. CSF levels of IL-8, IL-12p40, IL-17A, TNF-α, INF-γ and sera TNF-α were significantly higher among survivors, which indicates that this progressive shift in cytokine expression favouring a Th1 pattern is crucial in controlling cryptococcal infection and would be a prognostic marker in cryptococcal meningitis (Mora et al. 2015, Mora et al. 2017). In addition, the study of the cytokine profile of human peripheral blood mononuclear cells (PBMCs) of healthy individuals, after *in vitro* stimulation with heat-killed *C. gattii* and *C. neoformans* (40 different strains), demonstrated that clinical heat-killed *C. gattii* isolates induced a more pronounced inflammatory response with higher concentrations of pro-inflammatory cytokines (IL-1β, TNF-α and IL-6 and the Th17/22 cytokine IL-17 and IL-22) compared to other *Cryptococcus* species and non-clinical *C. gattii*, which is dependent on TLR4 and TLR9 as cellular receptors (Schoffelen et al. 2013).

By using vertebrate models, Garro et al. (2011a), Garro et al. (2011b) showed that in rats, peritoneal eosinophils can migrate into lymphoid organs to act as antigen-presenting cells of *C. neoformans* (strain 102/85) antigens, priming naïve and re-stimulating infected animals to induce T-cell and B-cell responses, and the development of a Th1 microenvironment with increased levels of nitric oxide (NO) synthesis and *C. neoformans*-specific immunoglobulin production, which all lead to a protective immune response against subsequent infection with fungus. Also in rats, the mechanisms involved in macrophage apoptosis promoted by cryptococcal glucuronoxylomannan (GXM) through NO generation showed to be novel immunomodulatory mechanisms that could contribute to limit inflammation during infection in these group of rodents (Chiapello 2017). Obtained from mice, the monoclonal antibodies (mAb) 4B3 were shown to stimulate phagocytosis of the *C. neoformans* (strain 028 LMIPK) by macrophages without fungicidal effect, thus favouring yeast dissemination and decreasing the survival of mice due to cryptococcal infection (Gato-Armas et al. 2006, Illnait-Zaragozi et al. 2011a).

In addition to cryptococcosis alone, mouse models have been also used to study the influence of or interaction between other fungi, bacterial and viral agents, and *C. neoformans* and *C. gattii* infections. Fungal-fungal specific interactions between *C. neoformans* and *H. capsulatum*, which co-exist in the environment, that can impact in virulence and disease were investigated. Co-infected mice showed to have significantly higher mortality than infection with *C. neoformans* (strain H99) or *H. capsulatum* alone, or acapsulated strains, as shown by higher pulmonary fungal burden in co-infected animals. Enhanced pellicle formation with a hybrid polysaccharide matrix with higher reactivity to GXM mAbs and increased resistance to phagocytosis and killing by macrophages was also observed, which together indicate that a microbial interaction involving the transfer of virulence traits may translate into enhanced lethality during mixed fungal infections (Cordero et al. 2016). Secondly, the influence of microbiota in the host response against *C. gattii* (strain L27/01) was demonstrated. Germ-free (GF) mice were more susceptible to infection, showing lower survival, higher fungal burden in the lungs and brain, increased behavioural changes, reduced levels of inflammatory cytokines (IFN-γ, IL-1β and IL-17), and lower NFκBp65 phosphorylation compared to conventional mice, which were associated with smaller yeast cells and polysaccharide capsules in the lungs, and less tissue damage. Moreover, macrophages from GF mice showed reduced ability to engulf, produce reactive oxygen species (ROS), and kill *C. gattii*. Restoration of microbiota in mice that received feces from conventional animals or administration with *Escherichia coli*, made mice more responsive to infection, which was associated with increased survival and higher levels of inflammatory mediators (Costa et al. 2016). More recently, the interaction between *C. gattii* infection (strain L27/01) and the concurrent infection with influenza A virus (IAV) strain H1N1 was studied. This co-infection resulted in a major increase in morbidity and mortality, with severe lung damage and a high brain fungal burden when mice were infected in the acute phase of influenza multiplication, indicating that IAV infection is a predisposing factor for severe disease and adverse outcomes in mice co-infected with *C. gattii*. IAV not only alters the host response to the yeast, leading to recruitment of significantly more neutrophils and macrophages into the lungs, but also induces the production of type 1 interferons (IFN-α4/β) and significantly reduces the levels of IFN-γ, which is associated with an impaired immune response. Reduced phagocytosis, killing of cryptococci and production of ROS by IAV-infected macrophages were also shown, leading to increased proliferation of the fungus within macrophages (Oliveira et al. 2017).

*In vitro*, the interaction between antibodies and the cryptococcal capsule, and the effect of protective and non-protective mAbs on *C. neoformans* H99 replication and the capsule’s physical properties have been also examined. Protective mAbs were shown to directly alter cell division by trapping and preventing the full release of newly budded cells. This effect is caused by an Ab-mediated increase in capsule stiffness, involving cross-linking of GXM molecules. The ability of mAbs to impair *C. neoformans* budding through changes in the capsule’s mechanical properties indicates a new, non-classical mechanism of Ab function, and presents important implications for understanding Ab-mediated immunity (Cordero et al. 2013). As the interaction between *C. neoformans* H99 and the amoebae is similar to that of the yeast-macrophage association, the participation of fungal virulence-associated structures with *Acanthamoeba castellanii* was evaluated by Rizzo et al. (2017). Fungal extracellular vesicles (EVs) and the GXM were shown to be internalised by *A. castellanii* with no impact on the viability of amoebal cells. However, EVs, but not free GXM, modulate antifungal properties of *A. castellanii* by inducing enhanced yeast survival. Phagocytosis of *C. neoformans* by amoebal cells and the pathogenic potential in a *Galleria mellonella* model are not affected by EVs, but previous interactions with *A. castellanii* rendered fungal cells more efficient in killing this invertebrate host, which all support the notion that interaction of *C. neoformans* with environmental predators results in enhanced virulence (Rizzo et al. 2017).

Both *in vivo* and *in vitro*, the role of mammalian β-galactoside-binding protein Galectin-3 (Gal-3) in *C. neoformans* H99 infection was also revealed. Gal-3 levels no only augments in human sera but also in spleen, lung, and brain tissues of infected mice. Gal-3-deficient mice are more susceptible to cryptococcosis than wild-type animals, as the first group present higher fungal burden and lower animal survival. *In vitro* experiments showed that Gal-3 also inhibits fungal growth and exerts a direct lytic effect on *C. neoformans* EVs, which could benefit the host (Almeida et al. 2017).

Regarding the research efforts targeting alternative antifungal strategies, Baronetti et al. (2006), Baronetti et al. (2011) showed that subcutaneous pretreatment of rats with heat killed cells of the *C. neoformans* strain 102/85 (HKC) emulsified in complete Freund adjuvant (CFA), promoted protection against an intraperitoneal challenge with viable *C. neoformans* var. *grubii* with significantly better clearance of yeasts from tissues, a lower concentration of GXM in serum and tissues, and better histopathological parameters compared to un-pretreated infected rats. Passive immunisation with a mAb to glucosylceramides (GlcCer), a lipid that is present at the fungal plasma membrane, cell wall, and secretory vesicles, and which induces antifungal antibodies and regulates the virulence of *C. neoformans* in animal infections, significantly reduced host inflammation and prolonged the survival of mice lethally infected with *C. neoformans* strain 24067 (Serotype D), revealing a potential therapeutic strategy to control cryptococcosis (Rodrigues et al. 2007). *In vitro*, mAb-based drugs have shown to be potentially a powerful alternative to standard antifungals, and although *C. neoformans* H99 was reported to be the least susceptible among other fungal species, it was found that wheat germ agglutinin (WGA), linked to the effector Fc region of murine IgG2a (WGA-Fc), was able to inhibit *in vitro* fungal growth, to increase phagocytosis by macrophages, as well as their antifungal functions (Liedke et al. 2017). Lastly, treatment of *C. neoformans* H99 cells with WGA followed by infection of mice also delayed mortality relative to animals infected with untreated fungal cells. Reduced brain colonisation by lectin-treated cryptococci was also observed. Blocking chitin-like oligomers also rendered yeast cells less efficient in their ability to associate with phagocytes. WGA did not affect fungal viability, but inhibited GXM release to the extracellular space and capsule formation. In WGA-treated yeast cells, genes that are involved in capsule formation and GXM traffic had their transcription levels decreased in comparison with untreated cells (Fonseca et al. 2013).

In this way, the wide variety of approaches and methodologies used in different laboratories from Argentina, Brazil and Cuba have globally contributed to understand particular issues related to the host-pathogen interactions in cryptococcosis.

*Laboratory data: Antimicrobial susceptibility of clinical and environmental isolates* - Most of the data in this topic have been generated on the numerous studies carried out in Brazil and Argentina. The studies have been focused on the methodology and interpretation of the phenotypic tests employed, the susceptibility data generated with clinical and environmental isolates and the study of resistance to some antifungals (Table IV).

**TABLE IV:** Latin American studies on the antifungal susceptibility of *Cryptococcus neoformans* and *C. gattii* clinical isolates: methodology, epidemiological cut-off values (ECV), susceptibility patterns, resistance and heteroresistance results

Country	Comment	Reference
Argentina	Susceptibility data for FCZ and AmB for *C. neoformans* isolates from HIV-positive patients	Arechavala et al. (2009)
	Correlation of ETest and Neo-Sensitabs diffusion assays with broth microdilution reference method (CLSI-M27-A2) for testing susceptibility of *C. neoformans* to AmB and FCZ	Ochiuzzi et al. (2010)
	*In vitro* activity of AmB B by time-kill curve methodology	Córdoba et al. (2011)
	International study of wild-type susceptibility endpoint distributions and epidemiological cutoff values for FCZ, ITZ, PCZ, and VCZ. Argentina, Brazil, Cuba, Mexico	Espinel-Ingroff et al. (2012a)
	Comparison of different in vitro tests to detect *C. neoformans* not susceptible to AmB	Córdoba et al. (2015)
	Susceptibility profile and epidemiological cut-off values of *C*. *neoformans* species complex from Argentina	Córdoba et al. (2016)
	*C. neoforman*s lanosterol 14-α-demethylase involved in fluconazole resistance in clinical isolates	Bosco-Borgeat et al. (2016)
Brazil	An international study of wild-type susceptibility endpoint distributions and epidemiological cutoff values for AmB and 5FC in *C. neoformans* and *C. gattii*	Espinel-Ingroff et al. (2012a)
	Susceptibility to antifungal agents and genotypes of Brazilian clinical and environmental *C. gattii* strains	Silva et al. (2012)
	Microbiological characteristics of clinical isolates of *Cryptococcus* spp. in Bahia, Brazil: molecular types and antifungal susceptibilities	Matos et al. (2012)
	Environmental isolation, biochemical identification, and antifungal drug susceptibility of *Cryptococcus* spp.	Teodoro et al. (2013)
	Susceptibility profile of clinical and environmental isolates of *C. neoformans* and *C. gattii*	Andrade-Silva et al. (2013)
	A flow cytometry method for testing the susceptibility of *Cryptococcus* spp. to AmB	Benaducci et al. (2015)
	*In vitro* susceptibility testing of AmB for *C. neoformans* variety *grubii* AFLP1/VNI and *C. gattii* AFLP6/VGII by CLSI and flow cytometry	Morales et al. (2015)
	Heteroresistance to ITZ and changes in the morphology and virulence of *C. gattii*	Ferreira et al. (2015)
	Antifungal susceptibility of clinical *C. deuterogattii* (AFLP6/VGII) isolates from Southern Brazil	Herkert et al. (2016)
	Resistance to FCZ and changes on virulence and morphological aspects of *C. neoformans* and *C. gattii*	Rossi et al. (2016)
	Antifungal susceptibility testing and genotyping characterization of *C. neoformans* and *C. gattii* isolates from HIV-infected patients	Figueiredo et al. (2016)
	Evaluation of antifungal combination against *Cryptococcus* spp.	Reichert-Lima et al. (2016)
	*C. neoformans* and *C. gattii* isolates from both HIV-infected and uninfected patients: antifungal susceptibility and outcome of cryptococcal disease	Nascimento et al. (2017)
	FCZ non-susceptible *C. neoformans*. Use of LAmB in AIDS patient	Santana et al. (2017)
	FCZ levels in serum and cerebrospinal fluid	Schiave et al. (2018)
	Time-kill curves studies with AmB against *C. neoformans*/*C*. *gattii* species complex clinical isolates	de Oliveira et al. (2017)
	Heteroresistance to FCZ in clinical and environmental strains of *C. neoformans* and *C. gattii*	Feliciano et al. (2017)
Cuba	*C. neoformans*, FCZ susceptibility	Fernández-Andreu et al. (1999)
	New azoles and *C. neoformans* Cuban clinical isolates	Illnait-Zaragozi et al. (2008)
	FCZ, VCZ susceptibility of Cuban isolates	Illnait-Zaragozi et al. (2009)
Venezuela	A review of *C. neoformans* ECVs	Ferrara et al. (2017)

AmB: amphotericin B; FCZ: fluconazole; ITZ: itraconazole; PCZ: posaconazole; VCZ: voriconazole.

*Methodology and interpretation of results* - Two reference methods have been used in Latin American studies to determine the minimal inhibitory concentration (MIC), the Clinical Laboratory Standard Institute (CLSI) microdilution method from M27-A3, and the EDef 7.2 from the European Committee on Antimicrobial Susceptibility Testing (EUCAST). Several additional techniques have been developed to simplify the standard ones, such as, time kill curves (TKC), E-Test strips, and a disk diffusion method (Ochiuzzi et al. 2010, Córdoba et al. 2015, de Oliveira et al. 2017). Recently, the use of flow cytometry (FC) was evaluated in two publications, which both showed a positive significant correlation (Benaducci et al. 2015, Morales et al. 2015). In one of them, the conclusion was that FC has an excellent correlation with the standard methods, and it is a reliable, fast, and safe method to test the susceptibility to AmB (Benaducci et al. 2015). In the other study, the antifungal susceptibility of 17 clinical isolates of *C. neoformans* and 18 *C. gattii* VGII, was analysed by both the microdilution method (CLSI M27-A3) and FC, showing that FC allowed to determine MIC for AmB in a 2 h incubation time, with MICs data obtained on the same day (Morales et al. 2015).

In three additional studies, the epidemiological cut-off values (ECV) for AmB, 5FC, FCZ, itraconazole (ITZ), posaconazole (PCZ) and voriconazole (VCZ), were established (Espinel-Ingroff et al. 2012a, Espinel-Ingroff et al. 2012b, Córdoba et al. 2016) and recently, a review done by the Venezuelan group was reported (Ferrara et al. 2017). The first two studies were conducted as a part of a collaborative international effort with the participation of Argentina, Brazil, Cuba, and Mexico (Espinel-Ingroff et al. 2012a, Espinel-Ingroff et al. 2012b). The rationale of this work was to propose clinical breakpoints for *C. neoformans* and *C. gattii*, according to the molecular types, which was not available, as such EVCs for AmB and 5FC, on basis of MICs (CLSI methodology) were determined. ECVs are commonly used to separate wild-type (WT) isolates from isolates with reduced susceptibility to antifungal drugs with probable acquired resistance mechanisms, are useful to monitor the emergence of strains with mutations that could lead to reduced antifungal susceptibility to antifungal drugs, however they should not be used as predictor of clinical outcome. In the study by Córdoba et al. (2016) with Argentinian isolates, the highest ECVs, which included ≥95% of the WT population studied, was observed for 5FC and FCZ (32 µg/mL each), values that were higher than those previously proposed by Espinel-Ingroff et al. (2012a), Espinel-Ingroff et al. (2012b).

*Clinical and environmental data* - The studies are presented in Table IV and the data is variable; hence, it is important to analyse each one of the articles’ data, separately (Arechavala et al. 2009, Illnait-Zaragozí et al. 2009, Matos et al. 2012, Silva et al. 2012, Andrade-Silva et al. 2013, Teodoro et al. 2013, Figueiredo et al. 2016, Herkert et al. 2016, Reichert-Lima et al. 2016, Nascimento et al. 2017).

As a general observation, clinical *C. gattii* strains are more resistant to FCZ in comparison to clinical *C. neoformans* strains. Otherwise, for AmB and 5FC no difference has been reported. Another general observation is that environmental isolates are more susceptible to antifungal drugs than clinical isolates.

It is important to mention the work by de Oliveira et al. (2017) describing the phenomenon of tolerance to AmB in some low-MIC strains of *C. neoformans* and *C. gattii*. Clinical studies are warranted to ascertain the relevance of this occurrence as a tool for guide treatment. Additionally, resistance to FCZ was studied by Bosco-Borgeat et al. (2016), in which they found that lanosterol 14-α-demethylase was involved in FCZ resistance in *C. neoformans* clinical isolates. The sequencing revealed the G1855A mutation in three isolates, resulting in the enzyme amino acid substitution G484S. These strains were isolated from two fluconazole-treated patients. Importantly, this mutation would not intervene in the susceptibility to the other widely use azoles, ITZ and VCZ.

*Novel compounds for treatment* - The synthesis of novel compounds has been explored by one research group in Colombia (Acosta et al. 2015, Ramírez et al. 2015, Montoya et al. 2016, Ramírez-Prada et al. 2017) and by two Brazilian groups (da Silva et al. 2016, Palanco et al. 2017) and their preliminary results look promising. Pyrazolonaphthyridines (Acosta et al. 2015), thiazole-based pyrimido[4,5-b] [1,4] diazepines (Ramírez et al. 2015), chalcones and N-aryl-2-pyrazolines (Montoya et al. 2016), and quinolone-based pyrazoles (Ramírez-Prada et al. 2017) have shown antifungal activity not only against *C. neoformans* but also against *Candida albicans*, with some of them showing fungicidal, rather than fungistatic activity, which makes them good antifungal candidates.

*Laboratory data: Early diagnosis in HIV patients* - Early diagnosis of cryptococcosis in HIV infected patients has been a topic of interest in Latin America since 1995 (Negroni et al. 1995, Lizarazo et al. 2002, Costa et al. 2013). In all, diagnosis was established with the determination of circulating antigen (CrAg) with the Latex test. Results of the studies were dissimilar but the importance of the CD4+ cells count to increase the sensitivity of the test was established. In 2011, a new CrAg lateral flow assay (CrAg LFA) was developed and preliminary experience in Colombia, Brazil, and Argentina (Escandón et al. 2013, Vidal et al. 2016, Frola et al. 2017) showed comparable results to that demonstrated globally (Huang et al. 2015, Wake et al. 2018). That is, a Point-of-Care Test (POCT) like CrAg LFA plays an important role in the early diagnosis of cryptococcosis in HIV patients, especially in resource limited settings. Inclusion of the CrAgLFA in the National Guides for managements of the patients with AIDS must be a compromise of the countries in the region without delay.

*Searching the environmental niche: From avian droppings to tree hollows* - The saprobic nature of the *Cryptococcus* spp. is a well-established fact. Infection with *Cryptococcus* spp. is invariably acquired by exposure to blastoconidia or basidiospores from an exogenous source and the host becomes an accident in the life cycle of the yeast. Consequently, studies on the natural reservoir of *C. neoformans* and *C. gattii* hold paramount interest even though they require a very extensive, programmed, coordinated and sequential field-work. In some Latin-American countries, this search has been conducted for many years. For this review, we will focus on the most recent studies (Table V).

**TABLE V:** Findings from recent Latin American environmental studies searching for the niche of *Cryptococcus neoformans* and *C. gattii*

Country	Sample		Species recovered		Studies		Reference
		
	Bird excreta	Tree detritus	*C.* *neoformans*	*C. gattii*	Phenotypic	Molecular	
Argentina		X	X	X	X	**VNI, VGI**	Refojo et al. (2009)
	X		X		X		Álvarez et al. (2010)
		X		X		**VGI, VGIII**	Mazza et al. (2013)
		X	X	X		**VNI, VGI**	Cattana et al. (2014)
Brazil		X	X	X	X	**VNI, VGII**	Costa et al. (2009)
	X		X		X	**VNI, VNII,** MATa	Ferreira-Paim et al. (2011)
	X	X	X		X	**VNI, VNII**	Andrade-Silva et al. (2012)
	X		X		X		Takahara et al. (2013)
			X	X	AS		Andrade-Silva et al. (2013)
	X		X	X	AS		Teodoro et al. (2013)
		X		X	X	**VGII**	Anzai et al. (2014)
		X		X	X	**VGII**	Brito-Santos et al. (2015)
		X	X		X	**VNI,** MATa	Castro e Silva et al. (2016)
	X	X	X	X	AS	**VNI, VGII**	Alves et al. (2016)
Chile	X		X		AS		González-Hein et al. (2010)
		X	X	X			Toro-Zúñiga et al. (2015)
Colombia	X	X	X	X	X	**VGIII**	Escandón et al. 2005
				X	X	**VGIII**	Escandón et al. (2010)
		X		X	X	**VNI,** **B, VGI, VGIII**	Firacative et al. (2011)
	X	X	X	X	X	**VNI, VGII, VGIII**	Mak et al. (2015)
		X	X		X	**VNI**	Noguera et al. (2015)
		X	X	X	X	**VNI, VGIII**	Vélez and Escandón (2017)
Cuba	X		X			Satellites	Illnait-Zaragozi et al. (2010a)
		X	*Cryptococcus* spp.				Illnait-Zaragozi et al. (2012)
Mexico	X		X	X		X	Canónico-Gonzáles et al. (2013)

AS: antifungal susceptibility.

The search for the habitat of these yeasts have been successfully achieved in many parts of the world, including Latin America, following Emmons’ findings (Emmons 1960) on the strong association of cryptococci with soil enriched with dry pigeon excreta. Following this, studies on the recovery of the yeasts, not only from pigeon droppings but also from other bird faeces, especially from captive birds, have been successful good in Latin America (Álvarez et al. 2010, González-Hein et al. 2010, Ferreira-Paim et al. 2011, Andrade-Silva et al. 2012, Takahara et al. 2013, Teodoro et al. 2013). Although most of the isolates therein recovered were identified as *C. neoformans*, VNI, mating type alpha, it is worth mentioning that in one study (Teodoro et al. 2013) *C. gattii* was recovered in 5.2% of samples. Globally, it is known that most of the isolates recovered from bird droppings are *C. neoformans* (Kwon-Chung et al. 2014), with the percentage of recovery varying from place to place from 2% up to 70%.

From the numerous studies done globally on the search for the fungus in avian droppings, it has been very well established that bird droppings shielded from direct sun and ultraviolet light are an excellent substrate for the growth of the yeasts because of their high urea concentrations that is converted to ammonium, and carbamate by the urease enzyme, which is one of the landmark enzymes of the genus.

On the other hand, by the end of the 80s, the natural habitat of *C. gattii* was still unknown, until 1990, when David Ellis and Tania Pfeiffer (Ellis and Pfeiffer 1990) published their seminal paper revealing that the habitat of *C. gattii* in Australia was related to *Eucalyptus camaldulensis* trees.

Following the Australian report, recovery of *C. gattii* from *E. camaldulensis* detritus was achieved initially in Mexico (Licea et al. 1999) and later in other Latin American countries. However, it is of utmost importance that the work done by Marcia Lazera and her group in Fiocruz, Rio de Janeiro, Brazil, as they demonstrated that the niche of *C. gattii* is not only related with *Eucalyptus* spp. but with an uncountable number of tree species, with isolates recovered especially from tree hollows and decayed woods (Lazera et al. 1998, Fortes et al. 2001). Importantly, their studies revealed as well that trees are also a habitat for *C. neoformans* (Lazera et al. 1996), and that sometimes both species are found in the same tree. Those findings encouraged a group in Colombia to search for the habitat of *C. gattii* in a variety of trees and, described, for the first-time, almond trees (*Terminalia catappa)* as the natural habitat of *C. gattii*, serotype C, VGIII (Callejas et al. 1998). Recently the serotype and molecular type of this species has been isolated form *Tipuana tipu* a native tree from Argentina (Mazza et al. 2013).

Following Lazera’s findings, tree hollows, decayed wood and soil samples have been described as the habitat of *C. gattii*, and *C. neoformans* in a plethora of indigenous and imported trees in some Latin-American countries, including: Argentina (Refojo et al. 2009, Mazza et al. 2013, Cattana et al. 2014), Brazil (Costa et al. 2009, Andrade-Silva et al. 2012, Anzai et al. 2014, Brito-Santos et al. 2015, Castro e Silva et al. 2016), Chile (Toro-Zúñiga et al. 2015), Colombia (Escandón et al. 2005, Escandón et al. 2010, Firacative et al. 2011, Mak et al. 2015, Noguera et al. 2015, Vélez and Escandón 2017), and Cuba (Illnait-Zaragozí et al. 2010a, Illnait-Zaragozí et al. 2012) (Table V). Some of the trees genus and species that are the reservoir of *C. gattii* as well as *C. neoformans* are: *E. camaldulensis*, *E. tereticornis*, *Eucalyptus* spp., *Acacia* spp., *Corymbia ficifolia*, *Phoenix* spp., *Tipuana tipui*, *Moquilea tomentosa*, *Cassia grandis*, *Ficus* spp*.*, *Croton* spp*.*, *Cedrus* spp*.*, *T. catappa* and *Tabebuia rosea*.

The rationale for the association of *C. neoformans* and *C. gattii* with decayed wood as a primary ecological niche for both species is that they are lignicolous, the so-called wood decay fungi, due to laccase (diphenoloxidase), an enzyme that oxidizes diphenolic substrates to form long polymers of melanin. Laccase provides protection to the fungus in the environment, and it is one of the most important and most studied virulence factors in *Cryptococcus* (Kwon-Chung et al. 2014).

Environmental isolates have been analysed with some phenotypic and molecular techniques. Antifungal susceptibility has been determined in some of them (Andrade-Silva et al. 2013, Teodoro et al. 2013, Firacative et al. 2016) and the results are quite variable. Andrade-Silva et al. (2013) reported that 6.2% of the environmental *C. neoformans* isolates were resistant to FCZ. Although in general, clinical isolates have lower susceptibility than environmental isolates to AmB and ITZ and environmental isolates have had lower susceptibility than clinical isolates to FCZ, VCZ, and KCZ, Teodoro et al. (2013) reported that all environmental *C. neoformans* isolates were susceptible to the antifungals tested. In addition, Firacative et al. (2016) reported that *C. gattii* VGIII environmental isolates were less susceptible to 5FC, PCZ, VCZ, ITZ and FCZ, and more susceptible to AmB, compared with clinical and veterinary isolates. Clinical isolates of *C. neoformans* were reported to be less susceptible to antifungal drugs than environmental isolates (Souza et al. 2010). Although the contribution of these studies is of great importance, more work needs to be done to have enough data for an analysis, especially for the correlation between the source of the isolates, clinical and environmental, and their antifungal susceptibility profiles.

As it was mentioned earlier, molecular studies have been done with some of the environmental isolates, indicated on Table III and Table V, and the importance of the studies done in Latin America was revealed when the global diversity of the *C. gattii* VGII isolates was studied, and the results provided evidence on the evolution of this pathogen in North American Pacific Northwest and the dispersal from South America, most likely from the Amazonia and the Northeast of Brazil, where *C. gattii* VGII strains have shown to be the most variable genetically (Engelthaler et al. 2014).

Many questions about the presence of the fungus in the environment were still without a complete answer, and one of them was related to the survival time of the blastoconidia in the samples. In this way, a couple of studies were carried out in Latin America, and the findings indicated that blastoconidia could remain viable from 45 days up to ten years (Álvarez et al. 2013, Escandón and Castañeda 2015). Efforts have been also made in the design of a culture medium, which use results appropriate for environmental studies to recover the fungus with a major efficiency from the environment (Silva et al. 2012, Álvarez et al. 2013). It is worth mentioning that the Staib culture medium supplemented with seeds of *Guizotia abyssinica* (niger seeds) is the medium of choice for environmental studies. These seeds provide the substrate for the laccase and the end product is melanin, which is associated with the cell walls and gives the colonies a characteristic brown pigment allowing for the differentiation from other yeast colonies.

Related to *C. gattii*, the pioneer environmental studies that have been done in Latin America and the ongoing ones with the assistance of molecular tools will allow to understand more clearly the expansion of the ecological niche of this species.

Particularly in Colombia, a study on ecological niche modelling was also used to forecast the distribution of cryptococcal species and to produce risk area maps for cryptococcal disease in the country (Mak et al. 2015). This study was conducted following the success of accurately identifying ecological niche areas of human and animal cases of *C. gattii* in British Columbia, Canada (Mak et al. 2010) and also complements the report on niche prediction of *C. neoformans* and *C. gattii* in Europe (Cogliati et al. 2017). As ecological niche modelling is a great tool to very likely predict where these pathogenic yeasts survive in the environment and therefore to identify areas with high risk of infection, it would be relevant to conduct such studies in all Latin-American countries, in order to increase awareness of this disease in regions at risk of environmental colonisation of *C. neoformans* and *C. gattii*.

*Concluding remarks* - The study of cryptococcosis and its etiological agents have been taking place in several Latin-American countries for several years and because of this, significant progress has been made on topics such as clinical epidemiology, laboratory identification and typing of cryptococcal strains, searching, and understanding the environmental niche, and recently, on the basic biology and population genetics of *C. neoformans* and *C. gattii*.

*Key points* - (i) globally, *C. neoformans* molecular type VNI, causes more than 90% of the cases of cryptococcosis in Latin America and the main risk factor for acquiring it (70-90%) is to be infected with HIV. *C. gattii*, VGII and VGIII, are also recovered, affecting otherwise healthy individuals; (ii) AmB and FCZ are the antifungal drugs of choice, since 5FC is unavailable in the region; (iii) laboratory diagnoses and species or molecular type identification are performed with the standardised protocols; (iv) determination of the antifungal susceptibility shows that in 90% of cases, both species are susceptible to the antifungals tested, namely: AmB, 5FC, FCZ, PCZ, VCZ, and ITZ. Particularly, *C. gattii* VGIII, serotype C isolates have shown decreased susceptibility to azoles, especially to FCZ; (v) molecular studies of clinical and environmental isolates show that in Latin America, *C. neoformans,* molecular type VNI, is not only predominant among the clinical cases, but also in the environment, followed by *C. gattii* molecular type VGII, with similar proportions in both clinical-, and environmental sources; (vi) a great amount of information is from the Latin American studies on the environmental niche, especially for *C. gattii*; (vii) the increasing application of genotyping methods in Latin America, has also greatly contributed to the global study of *C. neoformans* and *C. gattii*, to the recognition of their great inter-, and intra-species genetic diversity, to understand how these pathogenic yeasts have spread around the world and what is their population structure, and to better define some disease aspects of cryptococcosis, an important mycosis in the region.

## References

[B1] Abegg MA, Cella FL, Faganello J, Valente P, Schrank A, Vainstein MH (2006). Cryptococcus neoformans and Cryptococcus gattii isolated from the
excreta of psittaciformes in a southern Brazilian zoological
garden. Mycopathologia.

[B2] Acosta P, Butassi E, Insuasty B, Ortiz A, Abonia R, Zacchino SA (2015). Microwave-assisted synthesis of novel
pyrazolo[3,4-g][1,8]naphthyridin-5-amine with potential antifungal and
antitumor activity. Molecules.

[B3] Aguiar P, Pedroso RDS, Borges AS, Moreira TA, Araujo LB, Roder D (2017). The epidemiology of cryptococcosis and the characterization of
Cryptococcus neoformans isolated in a Brazilian University
Hospital. Rev Inst Med Trop Sao Paulo.

[B4] Almeida F, Wolf JM, Silva TA, DeLeon-Rodriguez CM, Rezende CP, Pessoni AM (2017). Galectin-3 impacts Cryptococcus neoformans infection through
direct antifungal effects. Nat Commun.

[B5] Álvarez C, Barbosa GG, Oliveira RV, Morales BP, Wanke B, Lazera MS (2013). Techniques for the detection of pathogenic Cryptococcus species
in wood decay substrata and the evaluation of viability in stored
samples. Mem Inst Oswaldo Cruz.

[B6] Álvarez C, Salim R, Runco R (2010). Presencia de Cryptococcus neoformans en excreta de palomas
urbanas en San Miguel de Tucumán - Argentina. Bol Micol.

[B7] Alves GS, Freire AK, Bentes AS, Pinheiro JF, Souza JV, Wanke B (2016). Molecular typing of environmental Cryptococcus neoformans/C.
gattii species complex isolates from Manaus, Amazonas,
Brazil. Mycoses.

[B8] Aminnejad M, Cogliati M, Duan S, Arabatzis M, Tintelnot K, Castaneda E (2016). Identification and characterization of VNI/VNII and novel
VNII/VNIV hybrids and impact of hybridization on virulence and antifungal
susceptibility within the C. neoformans/C. gattii species
complex. PLoS ONE.

[B9] Aminnejad M, Diaz M, Arabatzis M, Castañeda E, Lazera M, Velegraki A (2012). Identification of novel hybrids between Cryptococcus neoformans
var. grubii VNI and Cryptococcus gattii VGII. Mycopathologia.

[B10] Andrade-Silva L, Ferreira-Paim K, Mora DJ, Silva PR, Andrade AA, Lages-Silva E (2012). RAPD analysis with the primer L15996 of Brazilian clinical and
environmental Cryptococcus neoformans isolates. Mycopathologia.

[B11] Andrade-Silva L, Ferreira-Paim K, Mora DJ, Silva PR, Andrade AA, Araujo NE (2013). Susceptibility profile of clinical and environmental isolates of
Cryptococcus neoformans and Cryptococcus gattii in Uberaba, Minas Gerais,
Brazil. Med Mycol.

[B12] Andrade-Silva L, Ferreira-Paim K, Silva-Vergara ML, Pedrosa AL (2010). Molecular characterization and evaluation of virulence factors of
Cryptococcus laurentii and Cryptococcus neoformans strains isolated from
external hospital areas. Fungal Biol.

[B13] Anzai MC, Lazera MS, Wanke B, Trilles L, Dutra V, Paula DA (2014). Cryptococcus gattii VGII in a Plathymenia reticulata hollow in
Cuiaba, Mato Grosso, Brazil. Mycoses.

[B14] Arechavala A, Negroni R, Messina F, Romero M, Marín E, Depardo R (2017). Cryptococcosis in an infectious diseases hospital of Buenos
Aires, Argentina. Revision of 2041 cases: diagnosis, clinical features and
therapeutics. Rev Iberoam Micol.

[B15] Arechavala AI, Ochiuzzi ME, Borgnia MD, Santiso GM. [ (2009). Fluconazole and amphotericin B susceptibility testing of
Cryptococcus neoformans: results of minimal inhibitory concentrations
against 265 isolates from HIV-positive patients before and after two or more
months of antifungal therapy]. Rev Iberoam Micol.

[B16] Ávila D, Villalobos MA (2016). Perfil epidemiológico y respuesta terapéutica de la infección por
Cryptococcus sp. en pacientes de Costa Rica en el Hospital San Juan de Dios,
Período 2008-2012. Rev Clín Esc Med UCR - HSJD.

[B17] Ávila G, González G (2007). Algunas manifestaciones neurológicas del síndrome de
inmunodeficiencia adquirida (SIDA) en pacientes del Hospital Universitario
Hernando Moncaleano Perdomo de Neiva 2001-200. Acta Neurol Colomb.

[B18] Baronetti JL, Chiapello LS, Aoki MP, Gea S, Masih DT (2006). Heat killed cells of Cryptococcus neoformans var. grubii induces
protective immunity in rats: immunological and histopathological
parameters. Med Mycol.

[B19] Baronetti JL, Chiapello LS, Garro AP, Masih DT (2011). Treatment of rats with heat killed cells (HKC) of Cryptococcus
neoformans var. grubii induces cellular activation in spleen and lymphatic
nodes. Comp Immunol Microbiol Infect Dis.

[B20] Barriga G, Asumir C, Mercado NF (2005). Actualidades y tendencias en la etiología de las
meningoencefalitis causadas por hongos y bacterias
(1980-2004). Rev Latinoamer Patol Clin.

[B21] Bejar V, Tello M, Garcia R, Guevara JM, Gonzales S, Vergaray G (2015). Molecular characterization and antifungal susceptibility of
Cryptococcus neoformans strains collected from a single institution in Lima,
Peru. Rev Iberoam Micol.

[B22] Benaducci T, Matsumoto MT, Sardi JC, Fusco-Almeida AM, Mendes-Giannini MJ (2015). A flow cytometry method for testing the susceptibility of
Cryptococcus spp. to amphotericin B. Rev Iberoam Micol.

[B23] Boekhout T, Theelen B, Diaz M, Fell JW, Hop WC, Abeln EC (2001). Hybrid genotypes in the pathogenic yeast Cryptococcus
neoformans. Microbiology.

[B24] Bosco-Borgeat ME, Mazza M, Taverna CG, Cordoba S, Murisengo OA, Vivot W (2016). Amino acid substitution in Cryptococcus neoformans lanosterol
14-alpha-demethylase involved in fluconazole resistance in clinical
isolates. Rev Argent Microbiol.

[B25] Brito-Santos F, Barbosa GG, Trilles L, Nishikawa MM, Wanke B, Meyer W (2015). Environmental isolation of Cryptococcus gattii VGII from indoor
dust from typical wooden houses in the deep Amazonas of the Rio Negro
Basin. PLoS ONE.

[B26] Callejas A, Ordonez N, Rodriguez MC, Castaneda E (1998). First isolation of Cryptococcus neoformans var. gattii, serotype
C, from the environment in Colombia. Med Mycol.

[B27] Calvo BM, Colombo AL, Fischman O, Santiago A, Thompson L, Lazera M (2001). Antifungal susceptibilities, varieties, and electrophoretic
karyotypes of clinical isolates of Cryptococcus neoformans from Brazil,
Chile, and Venezuela. J Clin Microbiol.

[B28] Canessa JC, Cabrera D, Eskenazi J, Samalvides F (2011). Associated factors for in-hospital mortality in patients with
meningeal cryptococcosis and HIV infection at a local hospital in Lima,
Peru. World J AIDS.

[B29] Cangelosi D, De Carolis L, Trombetta L, Wainstein C (2009). Criptococosis meníngea asociada al SIDA. Análisis de los
pacientes varones HIV (+) con criptococosis meníngea internados en la Sala
11 del Hospital Francisco J Muñiz. Rev Assoc Med Argent.

[B30] Canónico-González Y, Adame-Rodriguez JM, Mercado-Hernández R, Arechiga-Carvajal ET (2013). Cryptococcus spp. isolation from excreta of pigeons (Columba
livia) in and around Monterrey, Mexico. Springerplus.

[B31] Carbia M, Perera P, Arteta Z, Cabeza E, Balleste R, Gezuele E (2017). Characterisation of Cryptococcus isolates in
Uruguay. Rev Iberoam Micol.

[B32] Cardoso PH, Baroni FA, Silva EG, Nascimento DC, Martins MA, Szezs W (2013). Feline nasal granuloma due to Cryptoccocus gattii type
VGII. Mycopathologia.

[B33] Casali AK, Goulart L, Rosa e Silva LK, Ribeiro AM, Amaral AA, Alves SH (2003). Molecular typing of clinical and environmental Cryptococcus
neoformans isolates in the Brazilian state Rio Grande do Sul. FEMS Yeast Res.

[B34] Castañeda A, McEwen J, Hidalgo M, Castaneda E. [ (2004). Cryptococcus spp. DNA extraction from environmental
samples]. Biomedica.

[B35] Castañeda E, Lizarazo J (2012). Protocolo de estudio y manejo de los pacientes con
criptococosis. Infectio.

[B36] Castro e Silva DM, Santos DC, Martins MA, Oliveira L, Szeszs MW, Melhem MS (2016). First isolation of Cryptococcus neoformans genotype VNI MAT-alpha
from wood inside hollow trunks of Hymenaea courbaril. Med Mycol.

[B37] Castro MR, Córdoba H (2014). Características clínicas y laboratoriales de la coinfeccion
VIH-SIDA y criptococosis meningea en el Hospital Clínico Viedma de
Cochabamba, Bolivia. Gac Med Bol.

[B38] Castro-Jiménez MA, Rey-Benito JR, Duque-Beltrán S, Pinilla-Guevara CA, Bello-Pieruccini S, Agudelo-Mahecha CM (2011). Diagnóstico de micosis oportunistas en pacientes con VIH/sida: un
estudio de casos en Colombia. Infectio.

[B39] Cattana ME, Fernández MS, Rojas FD, Sosa ML, Giusiano G (2015). Genotypes and epidemiology of clinical isolates of Cryptococcus
neoformans in Corrientes, Argentina. Rev Argent Microbiol.

[B40] Cattana ME, Sosa ML, Fernandez M, Rojas F, Mangiaterra M, Giusiano G (2014). Native trees of the Northeast Argentine: natural hosts of the
Cryptococcus neoformans-Cryptococcus gattii species complex. Rev Iberoam Micol.

[B41] Cattana ME, Tracogna MF, Fernández MS, Rey MCC, Sosa MA, Giusiano GE (2013). Genotyping of Cryptococcus neoformans/Cryptococcus gattii complex
clinical isolates from Hospital “Dr. Julio C. Perrando”, Resistencia city
(Chaco, Argentina). Rev Argent Microbiol.

[B42] Chiapello LS (2017). Mechanisms of immunosupression induced by cryptococcal capsular
polysaccharide.

[B43] Cicora F, Petroni J, Formosa P, Roberti J (2015). A rare case of Cryptococcus gattii pneumonia in a renal
transplant patient. Transpl Infect Dis.

[B44] Cogliati M, Puccianti E, Montagna MT, Donno A, Susever S, Ergin C (2017). Fundamental niche prediction of the pathogenic yeasts
Cryptococcus neoformans and Cryptococcus gattii in Europe. Environ Microbiol.

[B45] Cogliati M, Zani A, Rickerts V, McCormick I, Desnos-Ollivier M, Velegraki A (2016). Multilocus sequence typing analysis reveals that Cryptococcus
neoformans var. neoformans is a recombinant population. Fungal Genet Biol.

[B46] Concha-Velasco F, González-Lagos E, Seas C, Bustamante B (2017). Factors associated with early mycological clearance in
HIV-associated cryptococcal meningitis. PLoS ONE.

[B47] Conde-Pereira C, Rodas-Rodríguez L, Díaz-Paz M, Palacios-Rivera H, Firacative C, Meyer W (2015). Fatal case of polymicrobial meningitis caused by Cryptococcus
liquefaciens and Mycobacterium tuberculosis complex in a human
immunodeficiency virus-infected patient. J Clin Microbiol.

[B48] Cordero RJ, Liedke SC, de SAGR, Martinez LR, Nimrichter L, Frases S (2016). Enhanced virulence of Histoplasma capsulatum through transfer and
surface incorporation of glycans from Cryptococcus neoformans during
co-infection. Sci Rep.

[B49] Cordero RJ, Pontes B, Frases S, Nakouzi AS, Nimrichter L, Rodrigues ML (2013). Antibody binding to Cryptococcus neoformans impairs budding by
altering capsular mechanical properties. J Immunol.

[B50] Córdoba S, Afeltra J, Vitale RG (2011). Evaluation of the in vitro activity of amphotericin B by
time-kill curve methodology against large and small capsulate C. neoformans
isolates. Diagn Microbiol Infect Dis.

[B51] Córdoba S, Isla MG, Szusz W, Vivot W, Altamirano R, Davel G (2016). Susceptibility profile and epidemiological cut-off values of
Cryptococcus neoformans species complex from Argentina. Mycoses.

[B52] Córdoba S, Vivot W, Szusz W, Isla G, Davel G (2015). Comparison of different in vitro tests to detect Cryptococcus
neoformans not susceptible to amphotericin B. Mycopathologia.

[B53] Costa MC, Santos JR, Ribeiro MJ, Freitas GJ, Bastos RW, Ferreira GF (2016). The absence of microbiota delays the inflammatory response to
Cryptococcus gattii. Int J Med Microbiol.

[B54] Costa MM, Madeira L, Feitosa RN, Ishak MO, Ishak R, Silva SH (2013). Detection of Cryptococcus neoformans capsular antigen in
HIV-infected patients in the state of Para in the north of
Brazil. Curr HIV Res.

[B55] Costa SP, Lazera MS, Santos WR, Morales BP, Bezerra CC, Nishikawa MM (2009). First isolation of Cryptococcus gattii molecular type VGII and
Cryptococcus neoformans molecular type VNI from environmental sources in the
city of Belem, Para, Brazil. Mem Inst Oswaldo Cruz.

[B56] Silva AMD, Freitas VP, Conserva GA, Alexandre TR, Purisco SU, Tempone AG (2016). Bioactivity-guided isolation of laevicarpin, an antitrypanosomal
and anticryptococcal lactam from Piper laevicarpu
(Piperaceae). Fitoterapia.

[B57] Silva BK, Freire AK, Bentes AS, Sampaio IL, Santos LO, Santos MS (2012). Characterization of clinical isolates of the Cryptococcus
neoformans-Cryptococcus gattii species complex from the Amazonas state in
Brazil. Rev Iberoam Micol.

[B58] Silva EC, Guerra JM, Torres LN, Lacerda AM, Gomes RG, Rodrigues DM (2017). Cryptococcus gattii molecular type VGII infection associated with
lung disease in a goat. BMC Vet Res.

[B59] Dammert P, Bustamante B, Ticona E, Llanos-Cuentas A, Huaroto L, Chávez VM (2008). Treatment of cryptococcal meningitis in Peruvian AIDS patients
using amphotericin B and fluconazole. J Infect.

[B60] Darzé C, Lucena R, Gomes I, Melo A (2000). Características clínicas laboratoriais de 104 casos de
meningoencefalite criptocócica. Rev Soc Bras Med Trop.

[B61] Davel G, Abrantes R, Brudny M, Cordoba S, Rodero L, Canteros CE (2003). 1st environmental isolation of Cryptococcus neoformans var.
gattii in Argentina. Rev Argent Microbiol.

[B62] Davel G, Canteros CE (2007). Situación de las micosis en la República
Argentina. Rev Argent Microbiol.

[B63] Oliveira L, Silva-Santos DC, Martins MA, Szeszs MW, Melhem MSC (2017). Time-kill curves studies with amphotericin B against Cryptococcus
neoformans/C. gattii species complex clinical isolates. Current Fungal Infection Reports.

[B64] Santos WRA, Meyer W, Wanke B, Costa SPSE, Trilles L, Nascimento JLM (2008). Primary endemic Cryptococcosis gattii by molecular type VGII in
the state of Pará, Brazil. Mem Inst Oswaldo Cruz.

[B65] Dromer F, Mathoulin S, Dupont B, Laporte A (1996). Epidemiology of cryptococcosis in France: a 9-year survey
(1985-1993). French Cryptococcosis Study Group. Clin Infect Dis.

[B66] D’Souza CA, Kronstad JW, Taylor G, Warren R, Yuen M, Hu G (2011). Genome variation in Cryptococcus gattii, an emerging pathogen of
immunocompetent hosts. MBio.

[B67] Ellis DH, Pfeiffer TJ (1990). Natural habitat of Cryptococcus neoformans var.
gattii. J Clin Microbiol.

[B68] Emmons CW (1960). Prevalence of Cryptococcus neoformans in pigeon
habitats. Public Health Rep.

[B69] Engelthaler DM, Hicks ND, Gillece JD, Roe CC, Schupp JM, Driebe EM (2014). Cryptococcus gattii in North American Pacific Northwest:
whole-population genome analysis provides insights into species evolution
and dispersal. MBio.

[B70] Escandón P, Castañeda E (2015). Long-term survival of Cryptococcus neoformans and Cryptococcus
gattii in stored environmental samples from Colombia. Rev Iberoam Micol.

[B71] Escandón P, Bedout C, Lizarazo J, Agudelo CI, Tobon A, Bello S (2012). Cryptococcosis in Colombia: results of the national surveillance
program for the years 2006-2010. Biomedica.

[B72] Escandón P, Lizarazo J, Agudelo CI, Chiller T, Castañeda E (2013). Evaluation of a rapid lateral flow immunoassay for the detection
of cryptococcal antigen for the early diagnosis of cryptococcosis in HIV
patients in Colombia. Med Mycol.

[B73] Escandón P, Quintero E, Granados D, Huerfano S, Ruiz A, Castañeda E (2005). Isolation of Cryptococcus gattii serotype B from detritus of
Eucalyptus trees in Colombia. Biomedica.

[B74] Escandón P, Sánchez A, Firacative C, Castaneda E (2010). Isolation of Cryptococcus gattii molecular type VGIII, from
Corymbia ficifolia detritus in Colombia. Med Mycol.

[B75] Escandón P, Sánchez A, Martinez M, Meyer W, Castaneda E (2006). Molecular epidemiology of clinical and environmental isolates of
the Cryptococcus neoformans species complex reveals a high genetic diversity
and the presence of the molecular type VGII mating type a in
Colombia. FEMS Yeast Res.

[B76] Espinel-Ingroff A, Aller AI, Canton E, Castanon-Olivares LR, Chowdhary A, Cordoba S (2012a). Cryptococcus neoformans-Cryptococcus gattii species complex: an
international study of wild-type susceptibility endpoint distributions and
epidemiological cutoff values for fluconazole, itraconazole, posaconazole,
and voriconazole. Antimicrob Agents Chemother.

[B77] Espinel-Ingroff A, Chowdhary A, Cuenca-Estrella M, Fothergill A, Fuller J, Hagen F (2012b). Cryptococcus neoformans-Cryptococcus gattii species complex: an
international study of wild-type susceptibility endpoint distributions and
epidemiological cutoff values for amphotericin B and
flucytosine. Antimicrob Agents Chemother.

[B78] Feliciano LM, Ramos SDP, Szeszs MW, Martins MA, Bonfietti LX, Oliveira RA (2017). Heteroresistance to fluconazol in clinical and environmental
brazilian strains of Cryptococcus neoformans/C. gattii species
complex. Current Fungal Infection Reports.

[B79] Fernández-Andreu CM, Pimentel-Turino T, Martinez-Machin GF, González-Miranda M (1999). Determinación de la concentración mínima inhibitoria de
fluconazol frente a Cryptococcus neoformans. Rev Cubana Med Trop.

[B80] Fernández-Concepción O, Fernández-Novales C, Ariosa-Acuña MC, Fernández-Novales J (2003). Caracterización de un grupo de pacientes con criptococosis del
sistema nervioso central. Rev Neurol.

[B81] Ferrara G, Urdaneta E, Panizo MM, Alarcón V, García N, Moreno X (2017). Molecular characterization of Cryptococcus neoformans and Cryptococcus
gattii by PCR-RFLP in Venezuela.

[B82] Ferreira GF, Santos JR, Costa MC, Holanda RA, Denadai AM, Freitas GJ (2015). Heteroresistance to Itraconazole alters the morphology and
increases the virulence of Cryptococcus gattii. Antimicrob Agents Chemother.

[B83] Ferreira-Paim K, Andrade-Silva L, Fonseca FM, Ferreira TB, Mora DJ, Andrade-Silva J (2017). MLST-based population genetic analysis in a global context
reveals clonality amongst Cryptococcus neoformans var. grubii VNI isolates
from HIV patients in Southeastern Brazil. PLoS Negl Trop Dis.

[B84] Ferreira-Paim K, Andrade-Silva L, Mora DJ, Pedrosa AL, Rodrigues V, Silva-Vergara ML (2011). Genotyping of Cryptococcus neoformans isolated from captive birds
in Uberaba, Minas Gerais, Brazil. Mycoses.

[B85] Figueiredo TP, Lucas RC, Cazzaniga RA, Franca CN, Segato F, Taglialegna R (2016). Antifungal susceptibility testing and genotyping characterization
of Cryptococcus neoformans and gattii isolates from HIV-infected patients of
Ribeirao Preto, Sao Paulo, Brazil. Rev Inst Med Trop Sao Paulo.

[B86] Firacative C, Roe CC, Malik R, Ferreira-Paim K, Escandon P, Sykes JE (2016). MLST and whole-genome-based population analysis of Cryptococcus
gattii VGIII links clinical, veterinary and environmental strains, and
reveals divergent serotype specific sub-populations and distant
ancestors. PLoS Negl Trop Dis.

[B87] Firacative C, Torres G, Rodriguez MC, Escandon P (2011). First environmental isolation of Cryptococcus gattii serotype B,
from Cucuta, Colombia. Biomedica.

[B88] Fonseca FL, Guimarães AJ, Kmetzsch L, Dutra FF, Silva FD, Taborda CP (2013). Binding of the wheat germ lectin to Cryptococcus neoformans
chitooligomers affects multiple mechanisms required for fungal
pathogenesis. Fungal Genet Biol.

[B89] Fortes ST, Lazera MS, Nishikawa MM, Macedo RC, Wanke B (2001). First isolation of Cryptococcus neoformans var. gattii from a
native jungle tree in the Brazilian Amazon rainforest. Mycoses.

[B90] Freire AK, Bentes AS, Sampaio IL, Matsuura AB, Ogusku MM, Salem JI (2012). Molecular characterisation of the causative agents of
Cryptococcosis in patients of a tertiary healthcare facility in the state of
Amazonas-Brazil. Mycoses.

[B91] Frola C, Guelfand L, Blugerman G, Szyld E, Kaufman S, Cahn P (2017). Prevalence of cryptococcal infection among advanced HIV patients
in Argentina using lateral flow immunoassay. PLoS ONE.

[B92] Gaona-Flores VA, Campos-Navarro LA, Cervantes-Tovar RM, Alcala-Martinez E (2016). The epidemiology of fungemia in an infectious diseases hospital
in Mexico city: a 10-year retrospective review. Med Mycol.

[B93] Garro AP, Chiapello LS, Baronetti JL, Masih DT (2011b). Eosinophils elicit proliferation of naive and fungal-specific
cells in vivo so enhancing a T helper type 1 cytokine profile in favour of a
protective immune response against Cryptococcus neoformans
infection. Immunology.

[B94] Garro AP, Chiapello LS, Baronetti JL, Masih DT (2011a). Rat eosinophils stimulate the expansion of Cryptococcus
neoformans-specific CD4(+) and CD8(+) T cells with a T-helper 1
profile. Immunology.

[B95] Gato-Armas R, Martinez-Machin GF, Rodríguez-Sánchez H, Otero-González A, Sarracent-Pérez J, Illnait-Zaragozi MT (2006). Reconocimiento de células intactas de Cryptococcus neoformans por
el AcM 4B3 antiglucuronoxilomanano. Rev Cubana Med Trop.

[B96] González EC, Araúz AB, Rodríguez A (2013). Meningitis por Cryptococcus neoformans en pacientes con
SIDA. Rev Med Panama.

[B97] González GM, Casillas-Vega N, Garza-González E, Hernández-Bello R, Rivera G, Rodríguez JA (2016). Molecular typing of clinical isolates of Cryptococcus
neoformans/Cryptococcus gattii species complex from Northeast
Mexico. Folia Microbiol.

[B98] González-Hein G, González-Hein J, Jarabran MCD (2010). Isolation of Cryptococcus neoformans in dry droppings of captive
birds in Santiago, Chile. J Avian Med Surg.

[B99] Hagen F, Illnait-Zaragozi MT, Bartlett KH, Swinne D, Geertsen E, Klaassen CH (2010). In vitro antifungal susceptibilities and amplified fragment
length polymorphism genotyping of a worldwide collection of 350 clinical,
veterinary, and environmental Cryptococcus gattii isolates. Antimicrob Agents Chemother.

[B100] Hasimoto e Souza LK, Costa CR, Fernandes OF, Abrão FY, Silva TC, Treméa CM (2013). Clinical and microbiological features of cryptococcal
meningitis. Rev Soc Bras Med Trop.

[B101] Headley SA, Di Santis GW, Alcantara BK, Costa TC, Silva EO, Pretto-Giordano LG (2015). Cryptococcus gattii-induced infections in dogs from Southern
Brazil. Mycopathologia.

[B102] Herkert PF, Hagen F, Salvador GLO, Gomes RR, Ferreira MS, Vicente VA (2016). Molecular characterisation and antifungal susceptibility of
clinical Cryptococcus deuterogattii (AFLP6/VGII) isolates from Southern
Brazil. Eur J Clin Microbiol Infect Dis.

[B103] Herrera MR, Fuentes JJ, Godoy JP (2014). Criptococosis meningea en pacientes con VIH/SIDA.

[B104] Hu G, Liu I, Sham A, Stajich JE, Dietrich FS, Kronstad JW (2008). Comparative hybridization reveals extensive genome variation in
the AIDS-associated pathogen Cryptococcus neoformans. Genome Biol.

[B105] Huang HR, Fan LC, Rajbanshi B, Xu JF (2015). Evaluation of a new cryptococcal antigen lateral flow immunoassay
in serum, cerebrospinal fluid and urine for the diagnosis of cryptococcosis:
a meta-analysis and systematic review. PLoS ONE.

[B106] Igreja RP, Lazera MS, Wanke B, Galhardo MC, Kidd SE, Meyer W (2004). Molecular epidemiology of Cryptococcus neoformans isolates from
AIDS patients of the Brazilian city, Rio de Janeiro. Med Mycol.

[B107] Illnait-Zaragozi MT, Gato-Armas R, Martínez-Machín GF, Otero-González A, Sarracent-Pérez J, Rodríguez-Sánchez H (2011a). Efecto del anticuerpo monoclonal 4B3 en la infección experimental
por Cryptococcus neoformans. Rev Cubana Med Trop.

[B108] Illnait-Zaragozi MT, Hagen F, Fernández-Andreu CM, Martínez-Machin GF, Polo-Leal JL, Boekhout T (2011b). Reactivation of a Cryptococcus gattii infection in a cheetah
(Acinonyx jubatus) held in the National Zoo, Havana, Cuba. Mycoses.

[B109] Illnait-Zaragozi MT, Martinez GF, Curfs-Breuker I, Fernández CM, Boekhout T, Meis JF (2008). In vitro activity of the new azole isavuconazole (BAL4815)
compared with six other antifungal agents against 162 Cryptococcus
neoformans isolates from Cuba. Antimicrob Agents Chemother.

[B110] Illnait-Zaragozi MT, Martínez-Machin GF, Fernández-Andreu CM, Boekhout T, Meis JF, Klaassen CH (2010a). Microsatellite typing of clinical and environmental Cryptococcus
neoformans var. grubii isolates from Cuba shows multiple genetic
lineages. PLoS ONE.

[B111] Illnait-Zaragozi MT, Martínez-Machin GF, Fernández-Andreu CM, Hagen F, Boekhout T, Klaassen CH (2010b). Microsatellite typing and susceptibilities of serial Cryptococcus
neoformans isolates from Cuban patients with recurrent cryptococcal
meningitis. BMC Infect Dis.

[B112] Illnait-Zaragozi MT, Martínez-Machin GF, Fernández-Andreu CM, Perurena-Lancha MR, Theelen B, Boekhout T (2012). Environmental isolation and characterisation of Cryptococcus
species from living trees in Havana city, Cuba. Mycoses.

[B113] Illnait-Zaragozi MT, Meis JF, Martinez-Machin GF, Curfs-Breuker I, Fernández-Andreu CM, Perurena-Lancha MR (2009). In vitro susceptibility of isolated Cryptococcus strains to
fluconazole and voriconazole. Rev Cubana Med Trop.

[B114] Illnait-Zaragozi MT, Ortega-González LM, Hagen F, Martínez-Machin GF, Meis JF (2013). Fatal Cryptococcus gattii genotype AFLP5 infection in an
immunocompetent Cuban patient. Med Mycol Case Rep.

[B115] Jarvis JN, Harrison TS (2016). Forgotten but not gone: HIV-associated cryptococcal
meningitis. Lancet Infect Dis.

[B116] Klock C, Cerski M, Goldani LZ (2009). Histopathological aspects of neurocryptococcosis in HIV-infected
patients: autopsy report of 45 patients. Int J Surg Pathol.

[B117] Kwon-Chung KJ, Fraser JA, Doering TL, Wang Z, Janbon G, Idnurm A (2014). Cryptococcus neoformans and Cryptococcus gattii, the etiologic
agents of cryptococcosis. Cold Spring Harb Perspect Med.

[B118] Lamotte JA (2014). Caracterización de los pacientes en fase sida con infecciones del
sistema nervioso central. Medisan.

[B119] Lazera MS, Cavalcanti MA, Trilles L, Nishikawa MM, Wanke B (1998). Cryptococcus neoformans var. gattii - evidence for a natural
habitat related to decaying wood in a pottery tree hollow. Med Mycol.

[B120] Lazera MS, Pires FD, Camillo-Coura L, Nishikawa MM, Bezerra CC, Trilles L (1996). Natural habitat of Cryptococcus neoformans var. neoformans in
decaying wood forming hollows in living trees. J Med Vet Mycol.

[B121] Leal AL, Faganello J, Fuentefria AM, Boldo JT, Bassanesi MC, Vainstein MH (2008). Epidemiological profile of cryptococcal meningitis patients in
Rio Grande do Sul, Brazil. Mycopathologia.

[B122] Leimann BC, Koifman RJ (2008). Cryptococcal meningitis in Rio de Janeiro state, Brazil,
1994-2004. Cad Saude Publica.

[B123] Licea BA, Garza DG, Urbieta VF, Olivares RAC (1999). Isolation and characterization of Cryptococcus neoformans var.
gattii from samples of Eucalyptus camaldulensis in Mexico
city. Rev Iberoam Micol.

[B124] Liedke SC, Miranda DZ, Gomes KX, Goncalves JLS, Frases S, Nosanchuk JD (2017). Characterization of the antifungal functions of a WGA-Fc (IgG2a)
fusion protein binding to cell wall chitin oligomers. Sci Rep.

[B125] Limper AH, Adenis A, Le T, Harrison TS (2017). Fungal infections in HIV/AIDS. Lancet Infect Dis.

[B126] Lindenberg AS, Chang MR, Paniago AM, Lazéra MS, Moncada PM, Bonfim GF (2008). Clinical and epidemiological features of 123 cases of
cryptococcosis in Mato Grosso do Sul, Brazil. Rev Inst Med Trop Sao Paulo.

[B127] Lizarazo J, Castro F, Arco M, Chaves O, Peña Y (2006). Infecciones oportunistas del sistema nervioso central en
pacientes con VIH atendidos en el Hospital Universitario Erasmo Meoz de
Cúcuta (1995-2005). Infectio.

[B128] Lizarazo J, Chaves O, Peña Y, Escandón P, Agudelo CI, Castañeda E (2012). Comparación de los hallazgos clínicos y de supervivencia entre
pacientes VIH positivos y VIH negativos con criptococosis meníngea en un
hospital del tercer nivel. Acta Médica Colombiana.

[B129] Lizarazo J, Escandón P, Agudelo CI, Castañeda E (2014a). Cryptococcosis in Colombian children and literature
review. Mem Inst Oswaldo Cruz.

[B130] Lizarazo J, Escandón P, Agudelo CI, Firacative C, Meyer W, Castañeda E (2014b). Retrospective study of the epidemiology and clinical
manifestations of Cryptococcus gattii infections in Colombia from
1997-2011. PLoS Negl Trop Dis.

[B131] Lizarazo J, Linares M, Bedout C, Restrepo A, Agudelo CI, Castañeda E (2007). Results of nine years of the clinical and epidemiological survey
on cryptococcosis in Colombia, 1997-2005. Biomedica.

[B132] Lizarazo J, Peña Y, Chaves O, Omaña R, Huérfano S, Castañeda E (2002). Early diagnosis of Cryptococcosis and Histoplasmosis in patients
living with AIDS. Preliminary report. IQEN.

[B133] Loftus BJ, Fung E, Roncaglia P, Rowley D, Amedeo P, Bruno D (2005). The genome of the basidiomycetous yeast and human pathogen
Cryptococcus neoformans. Science.

[B134] Lomes NR, Melhem MS, Szeszs MW, Martins MA, Buccheri R (2016). Cryptococcosis in non-HIV/non-transplant patients: a Brazilian
case series. Med Mycol.

[B135] Lugarini C, Goebel CS, Condas LA, Muro MD, Farias MR, Ferreira FM (2008). Cryptococcus neoformans isolated from passerine and psittacine
bird excreta in the state of Parana, Brazil. Mycopathologia.

[B136] Luque L, Fraenza LB, Raga AJ (2017). Criptococosis e histoplasmosis diagnosticadas en pacientes con VIH/SIDA
en un periodo de 15 años. Webpage from Colegio de Bioquímicos de la
Provincia de Córdoba.

[B137] Mak S, Klinkenberg B, Bartlett K, Fyfe M (2010). Ecological niche modeling of Cryptococcus gattii in British
Columbia, Canada. Environ Health Perspect.

[B138] Mak S, Vélez N, Castaneda E, Escandón P, Group CES (2015). The fungus among us: Cryptococcus neoformans and Cryptococcus
gattii ecological modeling for Colombia. J Fungi.

[B139] Mantilla JC, Cárdenas N (2009). Hallazgos neuropatológicos de la infección por VIH-SIDA: estudio
de autopsias en el Hospital Universitario de Santander, Bucaramanga,
Colombia. Colomb Med.

[B140] Mascarenhas-Batista AV, Souza NM, Sacramento E (2013). Fatores prognósticos na meningite criptocócica em hospital de
referência para doenças infecciosas. Rev Baiana Saude Publica.

[B141] Matos CS, Andrade AS, Oliveira NS, Barros TF (2012). Microbiological characteristics of clinical isolates of
Cryptococcus spp. in Bahia, Brazil: molecular types and antifungal
susceptibilities. Eur J Clin Microbiol Infect Dis.

[B142] Matsumoto MT, Fusco-Almeida AM, Baeza LC, Melhem MS, Medes-Giannini MJ (2007). Genotyping, serotyping and determination of mating-type of
Cryptococcus neoformans clinical isolates from São Paulo state,
Brazil. Rev Inst Med Trop São Paulo.

[B143] Maziarz EK, Perfect JR (2016). Cryptococcosis. Infect Dis Clin North Am.

[B144] Mazza M, Refojo N, Bosco-Borgeat ME, Taverna CG, Trovero AC, Roge A (2013). Cryptococcus gattii in urban trees from cities in North-eastern
Argentina. Mycoses.

[B145] Medina N, Samayoa B, Lau-Bonilla D, Denning DW, Herrera R, Mercado D (2017). Burden of serious fungal infections in Guatemala. Eur J Clin Microbiol Infect Dis.

[B146] Mejía C, del Valle MJ (2012). Caracterización epidemiológica, clínica y terapéutica de pacientes con
diagnóstico de VIH/SIDA y meningitis por Cryptococcus neoformans durante el
período de enero 2006 - junio 2011 en el Hospital Roosevelt,
Guatemala.

[B147] Méndez-Tovar LJ, Mejía-Mercado JA, Manzano-Gayosso P, Hernández-Hernández F, López-Martínez R, Silva-González I (2016). Frecuencia de micosis invasivas en un hospital mexicano de alta
especialidad. Experiencia de 21 años. Rev Med Inst Mex Seguro Soc.

[B148] Meyer W, Aanensen DM, Boekhout T, Cogliati M, Diaz MR, Esposto MC (2009). Consensus multi-locus sequence typing scheme for Cryptococcus
neoformans and Cryptococcus gattii. Med Mycol.

[B149] Meyer W, Castañeda A, Jackson S, Huynh M, Castañeda E, Group ICS (2003). Molecular typing of IberoAmerican Cryptococcus neoformans
isolates. Emerg Infect Dis.

[B150] Meyer W, Mitchell TG (1995). Polymerase chain reaction fingerprinting in fungi using single
primers specific to minisatellites and simple repetitive DNA sequences:
strain variation in Cryptococcus neoformans. Electrophoresis.

[B151] Mónaco LS, Antabak NT (2008). Criptococosis en pacientes con SIDA: estudio de casos en el
Hospital Paroissien en el período 1996-2007. Rev Argent Microbiol.

[B152] Montoya A, Quiroga J, Abonia R, Derita M, Sortino M, Ornelas A (2016). Hybrid molecules containing a 7-Chloro-4-aminoquinoline nucleus
and a substituted 2-pyrazoline with antiproliferative and antifungal
activity. Molecules.

[B153] Mora DJ, Colombo ERC, Ferreira-Paim K, Andrade-Silva LE, Nascentes GA, Silva-Vergara ML (2012). Clinical, epidemiological and outcome features of patients with
cryptococcosis in Uberaba, Minas Gerais, Brazil. Mycopathologia.

[B154] Mora DJ, Ferreira-Paim K, Andrade-Silva LE, Bragine T, Rocha IH, Ribeiro BM (2017). Cytokine patterns in a prospective cohort of HIV-infected
patients with cryptococcal meningitis following initiation of antifungal and
antiretroviral therapy. PLoS ONE.

[B155] Mora DJ, Fortunato LR, Andrade-Silva LE, Ferreira-Paim K, Rocha IH, Vasconcelos RR (2015). Cytokine profiles at admission can be related to outcome in AIDS
patients with cryptococcal meningitis. PLoS ONE.

[B156] Morales BP, Trilles L, Bertho AL, Neves I, Oliveira RVC, Wanke B (2015). In vitro susceptibility testing of amphotericin B for
Cryptococcus neoformans variety grubii AFLP1/VNI and Cryptococcus gattii
AFLP6/VGII by CLSI and flow cytometry. Mycoses.

[B157] Moreira TA, Ferreira MS, Ribas RM, Borges AS (2006). Criptococose: estudo clínico-epidemiológico, laboratorial e das
variedades do fungo em 96 pacientes. Rev Soc Bras Med Trop.

[B158] Moretti ML, Resende MR, Lazéra MS, Colombo AL, Shikanai-Yasuda MA (2008). Consenso em criptococose - 2008. Rev Soc Bras Med Trop.

[B159] Nascimento E, Silva MEB, Martinez R, Kress MRZ (2014). Primary cutaneous cryptococcosis in an immunocompetent patient
due to Cryptococcus gattii molecular type VGI in Brazil: a case report and
review of literature. Mycoses.

[B160] Nascimento E, Vitali LH, Kress M, Martinez R (2017). Cryptococcus neoformans and C. gattii isolates from both
HIV-infected and uninfected patients: antifungal susceptibility and outcome
of cryptococcal disease. Rev Inst Med Trop São Paulo.

[B161] Negroni R, Cendoya C, Arechavala AI, Robles AM, Bianchi M, Bava AJ (1995). Detection of Cryptococcus neoformans capsular polysaccharide
antigen in asymptomatic HIV-infected patients. Rev Inst Med Trop São Paulo.

[B162] Noguera MC, Escandón P, Castañeda E (2015). Cryptococcosis in Atlantico, Colombia: an approximation of the
prevalence of this mycosis and the distribution of the etiological agent in
the environment. Rev Soc Bras Med Trop.

[B163] Noguera MC, Escandon P, Castañeda E (2017). Fatal Cryptococcus gattii genotype VGI infection in an
HIV-positive patient in Barranquilla, Colombia. Rev Inst Med Trop Sao Paulo.

[B164] Ochiuzzi ME, Santiso GM, Arechavala AI (2010). Correlation of Etest and Neo-Sensitabs diffusion assays on
Mueller-Hinton-methylene blue agar with broth microdilution reference method
(CLSI-M27-A2) for testing susceptibilities of Cryptococcus neoformans to
amphotericin B and fluconazole. Med Mycol.

[B165] Olivares LR, Martinez KM, Cruz RM, Rivera MA, Meyer W, Espinosa RA (2009). Genotyping of Mexican Cryptococcus neoformans and C. gattii
isolates by PCR-fingerprinting. Med Mycol.

[B166] Oliveira LVN, Costa MC, Magalhães TFF, Bastos RW, Santos PC, Carneiro HCS (2017). Influenza A virus as a predisposing factor for
Cryptococcosis. Front Cell Infect Microbiol.

[B167] Palanco AC, Singulani JL, Costa-Orlandi CB, Gullo FP, Lourencetti NMS, Gomes PC (2017). Activity of 3’-hydroxychalcone against Cryptococcus gattii and
toxicity, and efficacy in alternative animal models. Future Microbiol.

[B168] Pérez C, Hernández Y, Guzmán ME, Arias F, Nweihed L, Landaeta ME (2009). Estudio clínico-epidemiológico de la criptococosis en Venezuela,
años 1994-2003. Kasmera.

[B169] Perfect JR, Dismukes WE, Dromer F, Goldman DL, Graybill JR, Hamill RJ (2010). Clinical practice guidelines for the management of cryptococcal
disease: 2010 update by the infectious diseases society of
America. Clin Infect Dis.

[B170] Pinto VL, Pone MV, Pone SM, Campos JM, Garrido JR, Barros AC (2010). Cryptococcus gattii molecular type VGII as agent of meningitis in
a healthy child in Rio de Janeiro, Brazil: report of an autochthonous
case. Rev Soc Bras Med Trop.

[B171] Prado M, Silva MB, Laurenti R, Travassos LR, Taborda CP (2009). Mortality due to systemic mycoses as a primary cause of death or
in association with AIDS in Brazil: a review from 1996 to
2006. Mem Inst Oswaldo Cruz.

[B172] Rajasingham R, Smith RM, Park BJ, Jarvis JN, Govender NP, Chiller TM (2017). Global burden of disease of HIV-associated cryptococcal
meningitis: an updated analysis. Lancet Infect Dis.

[B173] Ramírez BC, Vega YC, Shepherd BE, Le C, Turner M, Frola C (2017). Outcomes of HIV-positive patients with cryptococcal meningitis in
the Americas. Int J Infect Dis.

[B174] Ramírez J, Svetaz L, Quiroga J, Abonia R, Raimondi M, Zacchino S (2015). Synthesis of novel thiazole-based
8,9-dihydro-7H-pyrimido[4,5-b][1,4]diazepines as potential antitumor and
antifungal agents. Eur J Med Chem.

[B175] Ramírez-Prada J, Robledo SM, Velez ID, Crespo MDP, Quiroga J, Abonia R (2017). Synthesis of novel quinoline-based 4,5-dihydro-1H-pyrazoles as
potential anticancer, antifungal, antibacterial and antiprotozoal
agents. Eur J Med Chem.

[B176] Raso TF, Werther K, Miranda ET, Mendes-Giannini MJ (2004). Cryptococcosis outbreak in psittacine birds in
Brazil. Med Mycol.

[B177] Refojo N, Perrotta D, Brudny M, Abrantes R, Hevia AI, Davel G (2009). Isolation of Cryptococcus neoformans and Cryptococcus gattii from
trunk hollows of living trees in Buenos Aires City,
Argentina. Med Mycol.

[B178] Reichert-Lima F, Busso-Lopes AF, Lyra L, Peron IH, Taguchi H, Mikami Y (2016). Evaluation of antifungal combination against Cryptococcus
spp. Mycoses.

[B179] Reséndiz MA, Velázquez G, Pérez J, Chávez L, Olvera JE (2008). Criptococosis cerebral: análisis de 29 casos en 23 años de
autopsias en el Hospital General de México. Patología.

[B180] Reviákina V, Panizo M, Dolande M, Selgrad S (2007). Diagnóstico inmunológico de las micosis sistémicas durante cinco
años 2002-2006. Rev Soc Ven Microbiol.

[B181] Ribeiro AM, Silva LK, Schrank IS, Schrank A, Meyer W, Vainstein MH (2006). Isolation of Cryptococcus neoformans var. neoformans serotype D
from Eucalyptus in South Brazil. Med Mycol.

[B182] Ribeiro MA, Ngamskulrungroj P (2008). Molecular characterization of environmental Cryptococcus
neoformans isolated in Vitoria, ES, Brazil. Rev Inst Med Trop São Paulo.

[B183] Rivera V, Gaviria M, Muñoz-Cadavid C, Cano L, Naranjo T (2015). Validation and clinical application of a molecular method for the
identification of Cryptococcus neoformans/Cryptococcus gattii complex DNA in
human clinical specimens. Braz J Infect Dis.

[B184] Rizzo J, Albuquerque PC, Wolf JM, Nascimento R, Pereira MD, Nosanchuk JD (2017). Analysis of multiple components involved in the interaction
between Cryptococcus neoformans and Acanthamoeba castellanii. Fungal Biol.

[B185] Rodrigues ML, Shi L, Barreto-Bergter E, Nimrichter L, Farias SE, Rodrigues EG (2007). Monoclonal antibody to fungal glucosylceramide protects mice
against lethal Cryptococcus neoformans infection. Clin Vaccine Immunol.

[B186] Romero JC, Mejía CR, Rodríguez JD, Gularte VM, Cárcamo MR, Samayoa AJ (2005). Criptococosis meníngea en pacientes VIH-SIDA, en Hospital Roosevelt de
Guatemala. Análisis de 110 casos: 1999-2004.

[B187] Rossi SA, Trevijano-Contador N, Scorzoni L, Mesa-Arango AC, Oliveira HC, Werther K (2016). Impact of resistance to fluconazole on virulence and
morphological aspects of Cryptococcus neoformans and Cryptococcus gattii
isolates. Front Microbiol.

[B188] Sánches S, Zambrano D, García M, Bedoya C, Fernández C, Illnait-Zaragozi MT (2017). Caracterización molecular de los aislamientos de Cryptococcus neoformans
de pacientes con HIV, Guayaquil, Ecuador Biomedica.

[B189] Sánchez S, Zambrano D, Martínez GF, Fernández CM, Illnait-Zaragozí MR (2016). Neurocriptococosis en el contexto de la infección con el VIH en
Guayaquil, Ecuador. Rev Cubana Med Trop.

[B190] Santana RC, Schiave LA, Quaglio ASS, Gaitani CM, Martinez R (2017). Fluconazole non-susceptible Cryptococcus neoformans,
relapsing/refractory cryptococcosis and long-term use of liposomal
Amphotericin B in an AIDS patient. Mycopathologia.

[B191] Schiave LA, Nascimento E, Vilar FC, Haes TM, Takayanagui OM, Gaitani CM (2018). Fluconazole levels in serum and cerebrospinal fluid according to
daily dosage in patients with cryptococcosis and other fungal
infections. Braz J Infect Dis.

[B192] Schoffelen T, Illnait-Zaragozi MT, Joosten LA, Netea MG, Boekhout T, Meis JF (2013). Cryptococcus gattii induces a cytokine pattern that is distinct
from other cryptococcal species. PLoS ONE.

[B193] Severo CB, Xavier MO, Gazzoni AF, Severo LC (2009). Cryptococcosis in children. Paediatr Respir Rev.

[B194] Siachoque N, Jewtuchowicz VM, Iovannitti C, Mujica MT (2010). CAP59 gene amplification in Cryptococcus neoformans and
Cryptococcus gattii directly from a yeast suspension. Rev Argent Microbiol.

[B195] Sierra A (2013). Meningitis criptocóccica. Rev Nac.

[B196] Silva DC, Martins MA, Szeszs MW, Bonfietti LX, Matos D, Melhem MS (2012). Susceptibility to antifungal agents and genotypes of Brazilian
clinical and environmental Cryptococcus gattii strains. Diagn Microbiol Infect Dis.

[B197] Soares EA (2015). Mortalidade por criptococose no Brasil (2000 a 2012).

[B198] Solar S, Diaz V, Rosas R, Valenzuela S, Pires Y, Diaz M (2015). Infección de sistema nervioso central por Cryptococcus gattii en
paciente inmunocompetente: desafíos diagnósticos y terapéuticos en un Centro
Terciario de Referencia de Paciente Internacional en Santiago de
Chile. INFOCUS.

[B199] Souto AC, Bonfietti LX, Ferreira-Paim K, Trilles L, Martins M, Ribeiro-Alves M (2016). Population genetic analysis reveals a high genetic diversity in
the Brazilian Cryptococcus gattii VGII population and shifts the global
origin from the Amazon rainforest to the semi-arid desert in the Northeast
of Brazil. PLoS Negl Trop Dis.

[B200] Souza LK, Souza AH, Costa CR, Faganello J, Vainstein MH, Chagas AL (2010). Molecular typing and antifungal susceptibility of clinical and
environmental Cryptococcus neoformans species complex isolates in Goiania,
Brazil. Mycoses.

[B201] Takahara DT, Lazera MS, Wanke B, Trilles L, Dutra V, Paula DA (2013). First report on Cryptococcus neoformans in pigeon excreta from
public and residential locations in the metropolitan area of Cuiabá, state
of Mato Grosso, Brazil. Rev Inst Med Trop São Paulo.

[B202] Teodoro VL, Gullo FP, Sardi JC, Torres EM, Fusco-Almeida AM, Mendes-Giannini MJ (2013). Environmental isolation, biochemical identification, and
antifungal drug susceptibility of Cryptococcus species. Rev Soc Bras Med Trop.

[B203] Toro-Zúñiga V (2015). Aislamiento presuntivo y caracterización de Cryptococcus
neoformans y Cryptococcus gattii desde árboles en la región de O’Higgins y
Maule, Chile. Bol Micol.

[B204] Torres RG, Etchebehere RM, Adad SJ, Micheletti AR, Ribeiro BM, Silva LE (2016). Cryptococcosis in acquired immunodeficiency syndrome patients
clinically confirmed and/or diagnosed at necropsy in a teaching hospital in
Brazil. Am J Trop Med Hyg.

[B205] Trilles L, Lazera M, Wanke B, Theelen B, Boekhout T (2003). Genetic characterization of environmental isolates of the
Cryptococcus neoformans species complex from Brazil. Med Mycol.

[B206] Trilles L, Lazera MS, Wanke B, Oliveira RV, Barbosa GG, Nishikawa MM (2008). Regional pattern of the molecular types of Cryptococcus
neoformans and Cryptococcus gattii in Brazil. Mem Inst Oswaldo Cruz.

[B207] Trilles L, Wang B, Firacative C, Lazera MS, Wanke B, Meyer W (2014). Identification of the major molecular types of Cryptococcus
neoformans and C. gattii by hyperbranched rolling circle
amplification. PLoS ONEe.

[B208] Tsujisaki RA, Paniago AM, Lima MS, Alencar DS, Spositto FL, Nunes MO (2013). First molecular typing of cryptococcemia-causing Cryptococcus in
Central-West Brazil. Mycopathologia.

[B209] van der Horst CM, Saag MS, Cloud GA, Hamill RJ, Graybill JR, Sobel JD (1997). Treatment of cryptococcal meningitis associated with the acquired
immunodeficiency syndrome. National Institute of Allergy and Infectious
Diseases Mycoses Study Group and AIDS Clinical Trials Group. N Engl J Med.

[B210] Vélez N, Escandón P (2017). Report on novel environmental niches for Cryptococcus neoformans
and Cryptococcus gattii in Colombia: Tabebuia guayacan and Roystonea
regia. Med Mycol.

[B211] Vidal JE, Gerhardt J, Miranda EJP, Dauar RF, Oliveira GS, Oliveira ACP (2012). Role of quantitative CSF microscopy to predict culture status and
outcome in HIV-associated cryptococcal meningitis in a Brazilian
cohort. Diagn Microbiol Infect Dis.

[B212] Vidal JE, Toniolo C, Paulino A, Colombo A, Martins MA, Meira CS (2016). Asymptomatic cryptococcal antigen prevalence detected by lateral
flow assay in hospitalised HIV-infected patients in São Paulo,
Brazil. Trop Med Int Health.

[B213] Wake RM, Britz E, Sriruttan C, Rukasha I, Omar T, Spencer DC (2018). High cryptococcal antigen titers in blood are predictive of
subclinical cryptococcal meningitis among HIV-infected
patients. Clin Infect Dis.

[B214] Williamson PR, Jarvis JN, Panackal AA, Fisher MC, Molloy SF, Loyse A (2017). Cryptococcal meningitis: epidemiology, immunology, diagnosis and
therapy. Nat Rev Neurol.

